# Nitrogen fixation rates increase with diazotroph richness in the global ocean

**DOI:** 10.1038/s41598-026-61132-2

**Published:** 2026-07-11

**Authors:** Dominic Eriksson, Damiano Righetti, Fabio Benedetti, Nicolas Gruber, Lucas Paoli, Guillem Salazar, Shinichi Sunagawa, Meike Vogt

**Affiliations:** 1 Environmental Physics, Institute of Biogeochemistry and Pollutant Dynamics, ETH Zurich, 8092 Zürich, Switzerland; 2 Centre for Ocean Life, Technical University of Denmark, 2800 Kgs. Lyngby, Denmark; 3 Department of Biology, Institute of Microbiology and Swiss Institute of Bioinformatics, ETH Zurich, 8093 Zürich, Switzerland

**Keywords:** Ecology, Ecology, Environmental sciences, Microbiology, Ocean sciences

## Abstract

Marine nitrogen fixation is a key process to support and maintain the ocean’s primary production, yet our knowledge of the distribution and diversity of the diazotrophic microbes that are capable of fixing nitrogen is very limited. Here, integrating microscopic and metagenomic data, we determine the biogeography and richness of the main diazotrophic taxa across the global ocean. Analyzing 22,000 records and 15 species, we deduce a latitudinal gradient in diazotroph richness, with higher richness to the tropics driven by temperature and nutrient levels. Cyanobacteria dominate in nutrient-poor gyres, while non-cyanobacterial diazotrophs thrive in nutrient-rich zones. Across the global ocean, diazotroph richness is found to correlate positively with nitrogen fixation rates, suggesting a positive biodiversity-ecosystem function relationship. While this relationship is robust to spatial autocorrelation and confounding environmental drivers, spatial dependence in the global datasets and potential unmeasured covariates may influence local-scale inferences. The findings suggest that positive biodiversity–ecosystem functioning relationships with implications for global biogeochemical cycling exist in marine plankton.

**Supplementary Information:**

The online version contains supplementary material available at 10.1038/s41598-026-61132-2.

## Introduction

The quantification of the link between species diversity and ecosystem processes (i.e., biodiversity-ecosystem functioning, BEF) is fundamental to understanding the role that diversity plays in ecosystem functions across various ecosystems^1,2^. Such BEF relationships are well-established for terrestrial ecosystems^2^, but evidence for their equivalent in marine planktonic systems remains scarce^3^. In aquatic systems, experimental studies have shown that mixtures of species frequently increase rates of ecosystem processes, such as productivity or consumption, compared to monocultures^3^. Such positive BEF relationships can arise, for example, through niche complementarity, where diverse communities utilize resources more efficiently and reduce competition. Another mechanism promoting a positive BEF relationship is selection, where certain high-performing species with advantageous traits contribute most to ecosystem functions. Understanding the primary mechanisms behind BEF relationships is important for predicting how biodiversity shifts associated with climate change affect nutrient cycling and ecosystem functions, especially in marine environments^4^.

Nitrogen-fixing microorganisms, known as diazotrophs, play a critical role in supplying bioavailable nitrogen that support marine primary production, especially in nutrient-poor regions^5,6^. By converting atmospheric nitrogen into fixed and thus bioavailable forms, diazotrophs fertilize the ocean. In particular, they resupply the marine fixed nitrogen reservoir by replacing nitrogen lost through denitrification, thus providing a crucial marine ecosystem function^7^.

The number of species of marine diazotrophs is relatively small, reflecting the high specificity and very conservative nature of the key metabolic process involving the main enzyme, nitrogenase^8,9^. Known groups of diazotrophs include both cyanobacterial organisms, such as *Trichodesmium* and a suite of unicellular cyanobacteria (e.g.: UCYN), and non-cyanobacterial organisms^10–12^. But recent discoveries of new nitrogenase iron protein (*nifH)* gene lineages suggest the existence of previously unrecognized diazotrophs, significantly expanding the known diversity^11–14^. However, data records about marine diazotrophs are sparse in general, and often biased^8^. As a result, there are substantial knowledge gaps in our understanding of the global diversity and biogeography of diazotrophs^13,14^, even at the rather high level of establishing, for example, the relative importance of cyano- versus non-cyanobacterial diazotrophs and what this implies for global nitrogen fixation. Previous work has analyzed the global distribution of marine diazotrophs and the biogeographical controls influencing them^15,16^, but no study has comprehensively investigated the relationship between diazotroph richness and marine nitrogen fixation on a global scale to date. This gap limits projections of how diazotroph diversity and marine nitrogen fixation might respond to future climate change^17^.

Traditionally, cyanobacterial diazotrophs, especially *Trichodesmium*, have been regarded as the dominant contributors to global marine nitrogen fixation^18,19^. While early estimates were largely based on geochemical approaches^20^ or extrapolation of *Trichodesmium* incubation studies^19^, community-level analyses that included the contributions from a diverse array of other diazotrophs have since expanded our understanding^11,21,22^. Despite this progress, global estimates of marine nitrogen fixation continue to diverge strongly, ranging between 85 and 238 Tg N yr^-1^^23–25^.

One reason for this large range in the estimated global marine nitrogen fixation rates are the rather different strategies with which marine diazotrophs accomplish the difficult task of breaking the triple bond of N_2._ While diazotrophic cyanobacteria have been shown to exhibit a range of nitrogen-fixing strategies, including diurnal and nocturnal fixation and the formation of oxygen-protective heterocysts^25^, far less is known about the ecological strategies of non-cyanobacterial diazotrophs, which are distributed widely in ocean ecosystems and occupy diverse niches across depths and oxygen gradients^13^. Notably, recent research has identified a non-cyanobacterial nitrogen-fixing symbiont, *‘Candidatus Tectiglobus diatomicola’*, which supplies fixed nitrogen to its diatom host in exchange for photosynthetically derived carbon^11^. In addition to such symbiotic relationships, particle-associated lifestyles have been recognized as a key ecological strategy for non-cyanobacterial diazotrophs, enabling nitrogen fixation in microoxic niches within marine particles^26–30^.

Several taxa have been identified as dominant contributors to marine biological nitrogen fixation, including cyanobacterial lineages such as *Trichodesmium*, *UCYN-A* sublineages, *Crocosphaera* (*UCYN-B*), Cyanothece-like *UCYN-C*, and the *HBD* clade^8,31^. These taxa are consistently detected across global oceanographic surveys and are among the most abundant and spatially widespread diazotrophs^8–10^. Although *nifH*-based surveys suggest that the total diversity of marine diazotrophs includes hundreds of phylotypes^13^, large-scale species richness patterns can be approximated by subsets of abundant and widespread, rather than rare species^32^ because these species provide the majority of empirical data across locations. Such abundant marine diazotrophs span diverse ecological strategies, from free-living unicells and colonial aggregates, to endosymbiotic or organelle-like associations^25,33^, making them representative focal groups for examining macroecological biodiversity gradients.

The differences in ecological strategies between the different diazotrophs may be a core reason why regions with the highest rates of marine nitrogen fixation may be the most taxonomically diverse. Under the premise of a positive BEF, each taxon contributes to nitrogen fixation via distinct ecological strategies. In addition, higher diversity may drive increased nitrogen fixation over longer temporal scales, such as annual cycles, with diverse taxa with complementary ecological strategies accumulating their contributions throughout the year.

In this study, we explore the global distribution and diversity of marine diazotrophs, encompassing both cyanobacterial and non-cyanobacterial taxa, by integrating traditional microscopy and molecular data (qPCR and metagenomics) into species distribution models (SDMs)^34^. We apply an ensemble approach which addresses model uncertainties associated with scarce and biased data distribution, and those arising from methodological choices such as environmental predictor selection, algorithm choice, and background selection (see Materials and Methods)^35,36^. Averaging monthly taxon-specific spatial distributions from these models, we estimate global diazotroph richness and assess its correlation with independent model-based and observations-based marine nitrogen fixation estimates^8,24,37–39^ on a mean annual scale. We then analyze beta diversity indices (i.e., species turnover and nestedness)^40^ to evaluate spatial patterns in community composition and their links with diazotroph richness. We show that global marine diazotroph richness exhibits a strong latitudinal gradient, with highest richness in the tropical oceans, driven primarily by temperature and nutrient availability. Diazotroph richness is positively correlated with marine nitrogen fixation rates (median Spearman’s ρ≥0.54), suggesting a robust BEF relationship at the macroecological scale. Environmental stratification analyses demonstrate that this coupling is not a simple artifact of shared abiotic gradients, as the association remains consistent even within narrow thermal and productivity windows. Our work suggests that positive BEF relationships associated with the nitrogen cycle exist in plankton, with consequences for ocean primary productivity.

## Results

### Biogeographic patterns of diazotroph richness

**Fig. 1:**
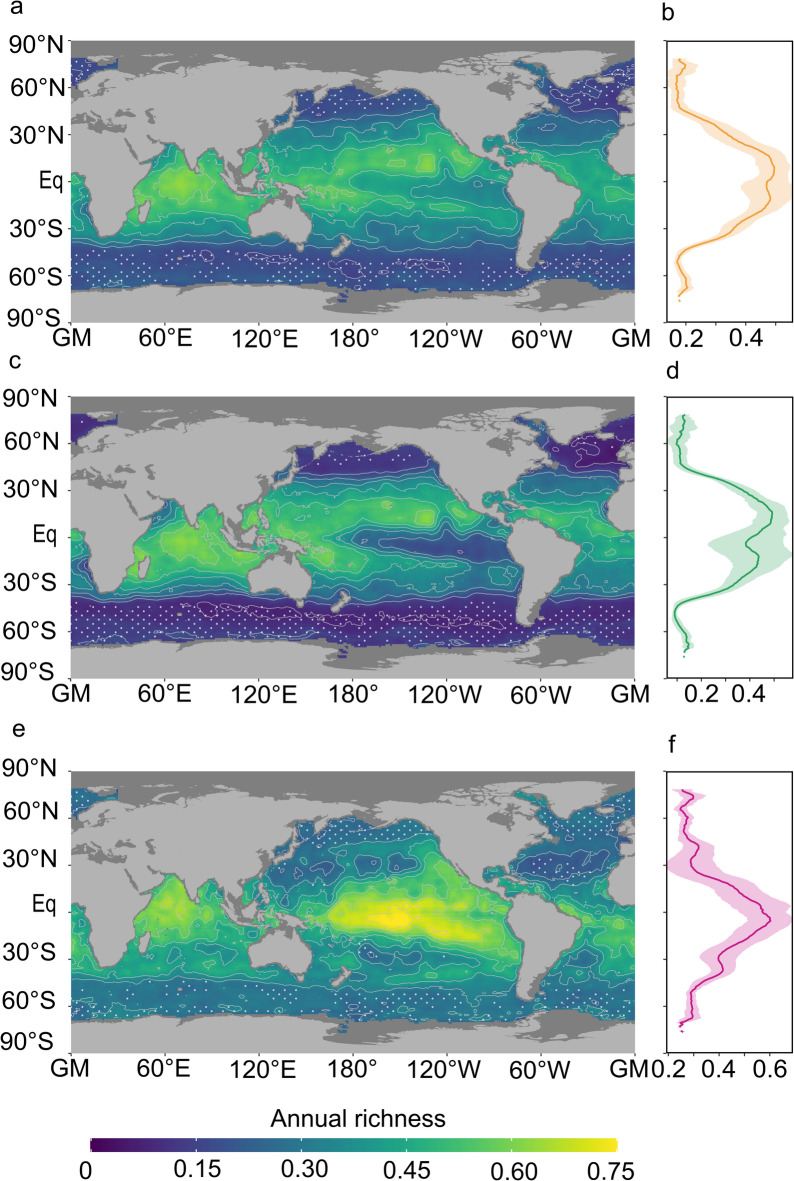
Global distribution of diazotroph richness. Maps show the mean annual diazotroph richness calculated as the average across 12 months for each 1° longitude by 1° latitude grid cell for (**a**) total (n = 15), (**c**) cyanobacterial (n = 9) and (**e**) non-cyanobacterial (n = 6) diazotrophs. White stipples indicate areas where the coefficient of variation is above the 70^th^ percentile, marking greater differences between ensemble members. Line plots show the latitudinal richness mean gradients (colored lines) across all 90 ensemble members for (**b**) total, (**d**) cyanobacterial and (**f**) non-cyanobacterial diazotrophs. Shading indicates ± 1 standard deviation across the 90 richness realizations per degree latitude.

To investigate the global mean annual pattern of marine diazotroph richness, we averaged the twelve monthly species richness distributions, each consisting of 90 ensemble members normalized by the respective number of taxa modeled. Richness was calculated by stacking corresponding presence-absence transformed member SDMs across species. Diazotrophs spanned totally 15 taxa that were successfully modeled (Supplementary Fig. 1), with 9 belonging to cyanobacterial (*Calothrix, Richelia, Richelia intracellularis, Trichodesmium erythreaum, Trichodesmium thiebautii, UCYN-A, UCYN-A1, UCYN-A2, UCYN-B* and *UCYN-C*) and 6 to non-cyanobacterial diazotrophs *(HBD-02* to -*06* and *Gamma-A*); we refer to these collectively as “species”. We find a latitudinal gradient in global normalized diazotroph species richness, which declines by half from the equator to the poles (Fig. 1a and b). Analyzed by latitude, normalized diazotroph richness reaches its maximum at ~15° north (mean ± sd = 0.54 ± 0.09), and ~16° south (mean = 0.54 ± 0.07) (Fig. 1b). At the equator, the normalized richness drops down to a longitudinally integrated mean value of 0.41 (± 0.07). This drop results from the low annual diazotroph richness predicted for the upwelling region in the equatorial Pacific Ocean. This latitudinal gradient in normalized diazotroph richness is consistent across all ensemble members, despite moderate variability among them (Fig. 1b, shading). Considering the fraction of grid cells where annual mean normalized diazotroph richness exceeds 0.5, the Indian Ocean ranks highest with 38% of grid cells, followed by the Pacific Ocean (24%) and the Atlantic (8%). Hotspots of elevated diazotroph richness are primarily found in the tropical Indian, and North Pacific gyre.

To address potential biases arising from mixing microscopy- and sequence-based presence data in our SDMs, we tested the sensitivity of our projections to methodological differences. Specifically, we reran the modeling pipeline using only microscopy-based or only sequence-based occurrence data for three taxa detectable by both methods: *Trichodesmium thiebautii*, *Trichodesmium erythraeum*, and *Richelia intracellularis*. The resulting global biogeographies showed strong spatial correlations between methods (Spearman’s ρ = 0.84, 95% CI[0.77, 0.89]; ρ = 0.68, 95% CI[0.60, 0.74]; ρ = 0.94, 95% CI[0.91, 095]; Supplementary Fig. 2), indicating that biogeographic patterns are largely consistent across data types, despite some variation for *T. erythraeum*. This agreement suggests that the integration of both microscopy and sequence observations may help reduce data limitations without substantially altering the overall richness patterns. However, we acknowledge that limited spatial overlap of methods, particularly among non-cyanobacterial diazotrophs, constrains broader inference.

Here, we modeled more than 18% of commonly described diazotrophs, comparable to the diversity of marine plankton captured by other recent modeling efforts (10–30%)^4,41^. Across all diazotrophs modeled in our study, we captured a broad range of life history strategies, including unicellular, colony-forming, symbiotic, heterotrophic, and autotrophic forms. To evaluate how our projected diversity patterns align with observed within-sample richness, we utilized the Ocean Microbiomics Database^41^, which captures a broader diazotroph diversity than our models, as it includes within-sample richness derived from metagenomic data. We compared the latitudinal richness patterns derived from our models with the observed within-sample richness by calculating Spearman correlation coefficients across 5° binned latitudinal richness gradients. This analysis revealed statistically significant correlations for total diazotroph richness (ρ = 0.70, p < 0.001), cyanobacterial diazotroph richness (ρ = 0.73, p < 0.001), and non-cyanobacterial diazotroph richness (ρ = 0.72, p < 0.001) (Supplementary Fig. 3). These results suggest that, despite modeling only a fraction of the total diazotroph richness contained by metagenomic datasets, our global patterns of diazotroph richness are likely representative.

To further assess the robustness of our global projected richness patterns, we iteratively removed one species at a time. We found that the clustering quality, as indicated by the Mean Silhouette Index, remained relatively stable (range: 0.43 to 0.45) until the removal of up to 9 species (~60% of the total; Supplementary Fig. 4). This finding underscores the robustness of the clustering structure despite a substantial reduction in modeled taxa.

The spread in normalized richness, a measure of incongruencies across the ensemble members, is generally higher at high latitudes, lowest within intermediate latitudes, and increasing again towards the tropics (Supplementary Fig. 5a). The spread is highest at ~70° northern and ~75° southern latitudes, and smallest at ~40° northern and ~35° southern latitudes. The tropical regions of the northern hemisphere show a higher spread than the tropical regions of the southern hemisphere. The global median of diazotroph richness across ensemble members ranges from 0.23 (IQR = 0.14) to 0.44 (IQR = 0.17) (Supplementary Fig. 6a).

The global distribution patterns of normalized richness differs between cyanobacterial and non-cyanobacterial diazotrophs in several ocean regions (Fig. 1c and e). Cyanobacterial diazotroph richness aligns closely with the latitudinal gradient observed for the total community (Spearman’s ρ = 0.89, p < 0.001), with notable declines in upwelling-influenced regions (Fig. 1d). In contrast, non-cyanobacterial diazotroph richness exhibits a latitudinal gradient increasing from poles to tropics without a significant drop at the equator, with these differences being particularly pronounced in the Pacific Ocean (Fig. 1e and f).

The spread across the ensemble members reveals distinct latitudinal trends in projection uncertainty (Supplementary Fig. 5b and c). Cyanobacterial normalized diazotroph richness shows low spread at high latitudes and increasing spread towards the tropics (Supplementary Fig. 5b), while non-cyanobacterial normalized diazotroph richness exhibits larger spread in higher latitudes (Supplementary Fig. 5c). These findings underscore the importance of considering spatial variability in projection uncertainty.

### Environmental drivers of diazotroph biogeography and diversity

To determine the most important environmental predictors for modeling individual diazotroph biogeographies based on presence/absences, we evaluated each predictor’s performance using adjusted D-squared (General Linear Model; GLM), adjusted R-squared (General Additive Model; GAM), and Out-Of-Bag Error (Random Forest; RF). The mean ranks of these metrics identified numerically relevant predictors for inclusion in our models (Supplementary Fig. 7a). Our analysis highlights sea surface temperature as the most influential predictor for diazotroph biogeography, with nitrate and phosphate concentrations following in second and third rank, respectively (Supplementary Fig. 7a). Specifically, the spatial distribution of cyanobacterial taxa exhibited stronger associations with N* (excess concentration of nitrate in relation to the redfield ratio), Si* (the ratio of nitrate to silicic acid), and photosynthetic active radiation (PAR), while the distribution of non-cyanobacterial diazotrophs showed closer ties to chlorophyll-a, phosphate, and nitrogen concentrations (Supplementary Fig. 7b), indicating a preference for more productive regions. Regarding diazotroph richness, sea surface temperature explains most of the variation in total diazotroph richness (Adj. R-squared = 0.87), with nutrients such as nitrate (Adj. R-squared = 0.78) and phosphate (Adj. R-squared = 0.68) ranking second and third, reflecting their importance in shaping individual taxa distributions (Supplementary Fig. 7).

### Diazotroph richness and marine nitrogen fixation

**Fig. 2:**
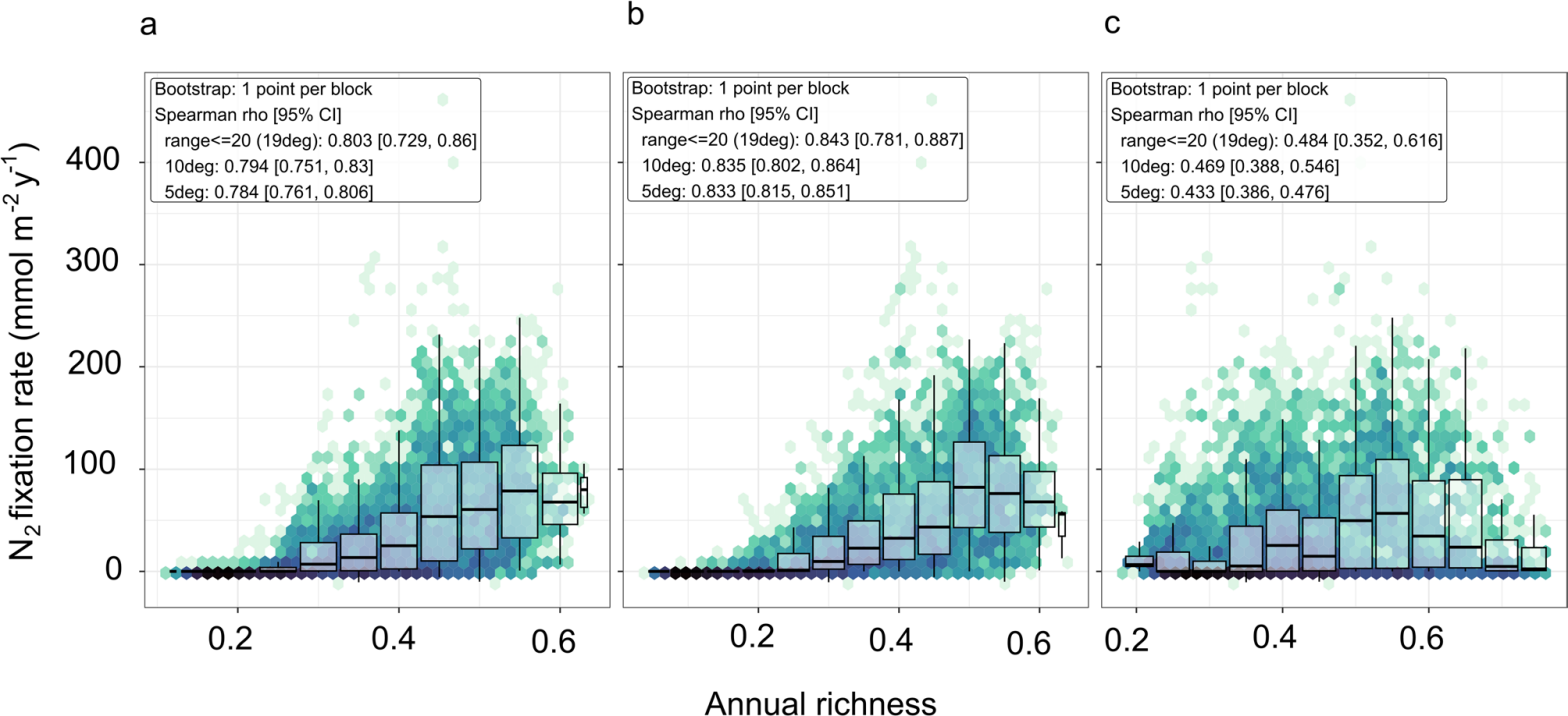
Global relationships between predicted annual diazotroph richness and marine nitrogen fixation rates. Panels evaluate the biodiversity–ecosystem functioning relationship across three diazotroph richness groups: (**a**) total, (**b**) cyanobacterial, and **c** non-cyanobacterial annual normalized richness, using the nitrogen fixation estimate from Wang et al.^24^ (mmol N m⁻^2^ y⁻^1^). Colors in each panel display the density of paired 1° × 1° grid cell values, using hexagonal bins. Insets provide a statistical assessment of the relationship, reporting the median Spearman’s ρ and 95% confidence intervals derived from one-point-per-block bootstrap summaries across three spatial block scenarios: a model-specific variogram range capped at 20°, and fixed 10° and 5° blocks.

To test whether a positive BEF relationship exists between normalized diazotroph richness and marine nitrogen fixation, we analyze the correlation between different spatially explicit estimates of marine nitrogen fixation rates^24,37^ (Supplementary Fig. 8) and our normalized diazotroph richness for total, cyanobacterial, and non-cyanobacterial diazotroph richness (Fig. 2).

We first related our predicted richness to oceanwide nitrogen fixation estimates from Wang et al.^24^, a model-based product, and Tang et al.^37^, a machine-learning-based extrapolation of *in situ* measurements. We find that rates of marine nitrogen fixation increase with total normalized diazotroph richness across all three nitrogen fixation datasets (median Spearman’s ρ ≥ 0.80, 95% CI [0.73,0.86]; Supplementary Fig. 9), after accounting for spatial autocorrelation. This positive BEF relationship is consistent across the majority of individual ensemble members for total and cyanobacterial richness (ensemble member range: ρ_min​_=0.06, ρ_max_​=0.88), though we observe broader variance and instances of negative correlations within the non-cyanobacterial richness group (ensemble member range: non-cyanobacterial richness: ρ_min_​=−0.57, ρ_max​_ = 0.82).

To evaluate the robustness of the identified BEF relationship across different ecological contexts, we tested whether a positive trend persists when the influence of dominant environmental drivers is constrained. Our results demonstrate that the positive association between richness and nitrogen fixation is not merely a byproduct of broad-scale global gradients (Supplementary Fig. 10). When the dataset is partitioned into discrete 2°C sea surface temperature bins, the correlation persists across the 20–28°C range (Supplementary Fig. 10a-c). For cyanobacterial richness, we observed median Spearman’s ρ values ranging from 0.28 to 0.51, with the strongest correlation (median ρ=0.51, n=3,015) occurring in the 24–26°C bin, a core thermal niche for many productive diazotrophs (Supplementary Fig. 10b). Furthermore, the relationship remains consistent across the global productivity gradient, persisting in the most oligotrophic waters (net primary production < 200 mg C m⁻^2^ d⁻^1^; median ρ=0.43, n=3,336; Supplementary Fig. 10d-f). The stability of these correlations across varying bin sizes (Supplementary Fig. 11) and environmental windows suggest that the BEF signal is an ecologically consistent feature of the diazotroph community that is not captured by temperature or bulk productivity alone.

A notable feature of the global BEF relationship is the emergence of a plateau in fixation rate at higher species richness for both cyanobacterial and total diazotrophs (Fig. 2). This saturating pattern is most pronounced for non-cyanobacterial and total diazotroph richness. For cyanobacteria, the relationship remains more linear across a broader range of nitrogen fixation rates, particularly when compared against the Wang et al.^24^ product. In areas of high richness, nitrogen fixation reaches a rather stable maximum, where further increases in species numbers are not associated with further increases in fixation rate.

To examine the robustness of the identified BEF relationship, we used *in situ* measurements of marine nitrogen fixation^8,38,39^ (Supplementary Fig. 12a) aggregated to different spatial extents in a temporally matched monthly analysis (Supplementary Fig. 12b-m). We averaged the available daily *in situ* nitrogen fixation measurements within spatial grid cells at monthly climatological resolution, retaining cells with at least two observations to minimize the influence of single-event stochasticity. These monthly *in situ* averages were paired with our corresponding monthly model projections of diazotroph richness. To ameliorate the high noise-to-signal ratio characteristic of sparse field data, we evaluated these correlations across a range of spatial grid cell resolutions, from 1° x 1° to 20° x 20°, using a spatial block bootstrap procedure to address potential pseudoreplication and autocorrelation.

Our analysis reveals a distinct scale-dependency in the strength of the BEF. While the relationships are shallow at 1° resolution, significant and steeper relationships emerged at higher data aggregation levels. At the 20° x 20° aggregation scale, we observed a statistically significant positive correlation for cyanobacterial richness (Spearman’s ρ=0.27, 95% CI [0.07,0.46]; Supplementary Fig. 12) and a positive, though non-significant, trend for total diazotroph richness (Spearman’s ρ=0.19, 95% CI [−0.03,0.38]; Supplementary Fig. 12k). In contrast, non-cyanobacterial richness showed no significant relationship with *in situ* rates at this scale (Spearman’s ρ=−0.02, 95% CI [−0.21,0.15]; Supplementary Fig. 12m). These results suggest that while individual snapshots of nitrogen fixation rate are influenced by environmental stochasticity or measurement variability, the diversity-functioning relationship is a persistent emergent property that becomes detectable when integrating data at coarser ecosystem-level resolution.

To understand which ecological mechanisms might drive the suggested positive BEF relationship between normalized diazotroph richness and marine nitrogen fixation rates, we first analyzed how differences in diazotroph community composition shape the global latitudinal normalized diazotroph richness gradient. Specifically, we calculated the beta ratio, which measures the relative contribution of two processes to differences in community composition: nestedness and turnover (Supplementary Fig. 13b and c). Nestedness refers to changes in community composition driven by differences in richness, where communities with fewer species are subsets of those with more species. Turnover, on the other hand, reflects the replacement of species by new ones across communities. A beta ratio greater than 0.5 indicates that differences in community composition are more strongly influenced by nestedness than by species turnover.

**Fig. 3:**
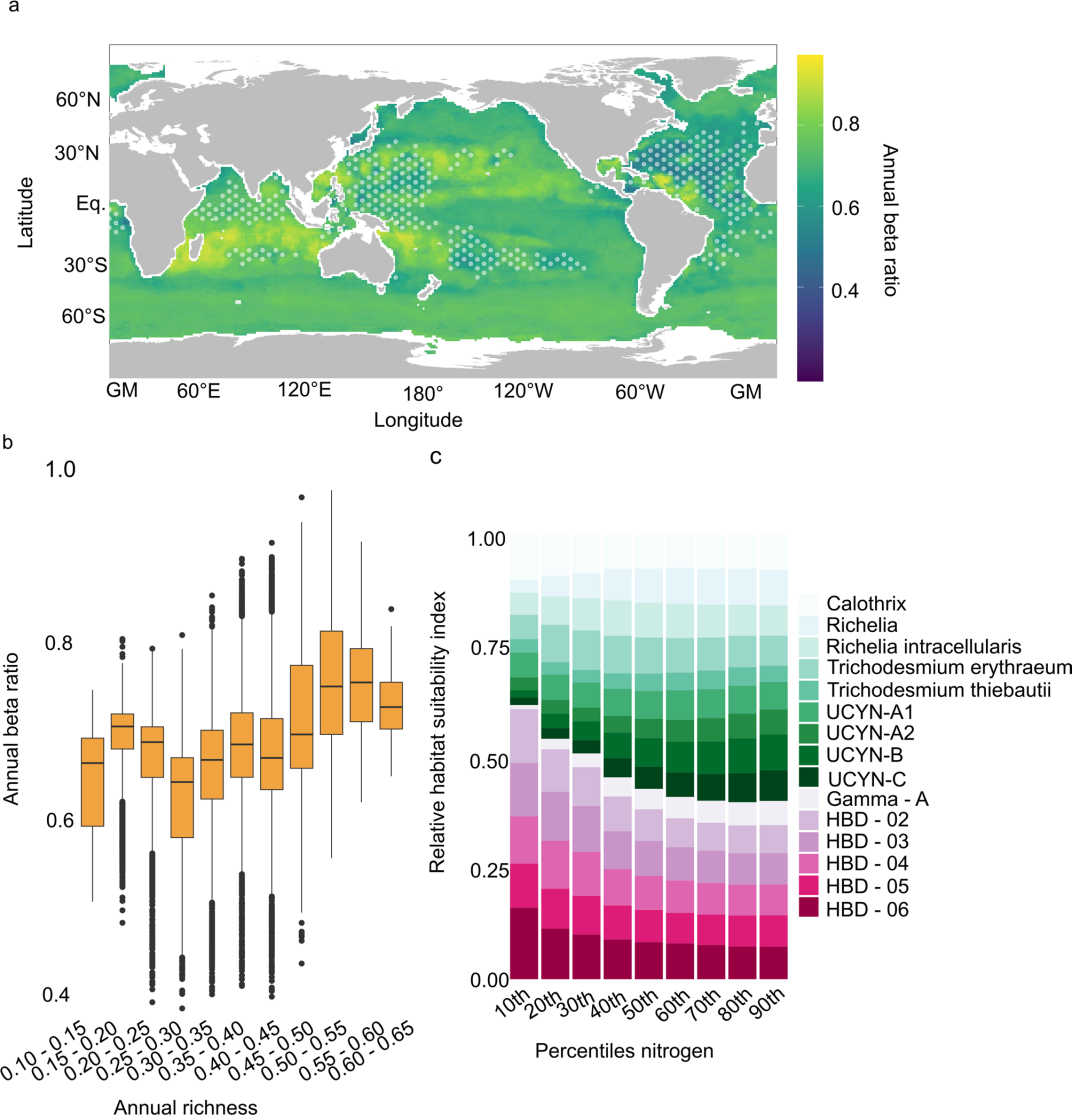
Global annual beta ratio distribution. (**a**) Global map of the annual beta ratio (calculated as nestedness/Jaccard) computed across 90 richness ensemble members, at 1° longitude by 1° latitude pixel resolution. A value of > 0.5 indicates that the nestedness is the major contributor underlying the change in community composition. (**b**) Boxplots of the annual beta ratio (y-axis) and the annual richness (x-axis). The bins illustrate normalized richness intervals, spanning a width of 0.05. (**c**) The stacked bar chart depicts the average habitat suitability indices for individual diazotroph taxa, with cyanobacterial taxa represented in green and non-cyanobacterial taxa in purple. The y-axis illustrates the relative proportion of mean annual habitat suitability. The x-axis represents locations ranked from lowest (10^th^ percentile) to highest (90^th^ percentile) annual nitrogen fixation rates according to Wang et al.^24^.

On a global scale, the beta ratio exceeds 0.5 in most ocean basins, with the notable exception of the North Atlantic (Fig. 3a). The decreasing beta ratio with decreasing richness from tropical to polar oceans, suggests that the global richness gradient is primarily driven by the progressive loss of certain taxa at higher latitudes, rather than by their replacement within the community.

To better illustrate the relationship between individual diazotroph distributions and global nitrogen fixation rates, we use the habitat suitability index (HSI), which quantifies the likelihood of occurrence based on the probabilistic SDM projections. We calculated the average HSI for each modeled taxon across marine regions ranked by their annual nitrogen fixation rates^24^, ranging from areas with the highest nitrogen fixation rates (above the 90^th^ percentile) to the lowest nitrogen fixation rates (below the 10^th^ percentile) (Fig. 3c). The relative averaged HSI for all cyanobacterial diazotrophs, except the genus *Calothrix*, is lower in regions with low annual nitrogen fixation rates. Meanwhile, non-cyanobacterial diazotrophs exhibit higher relative averaged HSI in regions with low nitrogen fixation rates. These diverging HSI patterns reinforce the observation that cyanobacterial richness is most strongly aligned with regions of high marine nitrogen fixation, whereas non-cyanobacterial diversity appears more decoupled from these functional hotspots.

The beta ratio estimates exhibit much greater variability across ensemble members in comparison to species richness (Supplementary Fig. 14). The majority of the ensemble members (79 out of 90) agree on nestedness being the major factor underlying the global richness gradient. We acknowledge that a part of the dominant role of nestedness could be due to species succession in time and conservative thresholds used when converting the habitat suitability index to presences. Analyzing changes in community composition at monthly scale found that community composition is most stable within tropical regions where diazotroph richness is highest (Supplementary Fig. 15).

## Discussion

The integration of classical microscopy-based and sequence-based (qPCR and metagenomic) data sources allowed us to model the biogeography of 15 diazotroph taxa (Supplementary Fig. 1) spanning a diverse range of functional traits and trophic strategies. This includes single-celled and colony-forming morphologies, as well as photoautotrophic and heterotrophic strategies, including non-cyanobacterial diazotrophs for which global model-based biogeographies have previously been data-limited (Supplementary Fig. 16). In accordance with previous SDM-based studies relying on plankton occurrences^9,35^, as well as studies based on metagenomic surveys^10,13^, we find diazotroph richness to be highest in tropical and subtropical regions and to decrease towards the poles (Fig. 1). This pattern aligns with the diversity distributions observed in other plankton functional groups and higher trophic levels^4,36^, suggesting that the key environmental drivers influencing species diversity, such as temperature and nutrient availability, are shared across a broad range of marine clades globally^4,36,42^. Our models also reveal the Indian Ocean as a hotspot for diazotroph richness. Considering the fraction of grid cells where mean diazotroph richness exceeds 0.5, the Indian Ocean ranks highest, with 38% of its grid cells. Although it is geographically smaller than the Pacific and Atlantic basins, its complex biophysical gradients and dynamic seasonal forcing may enhance habitat heterogeneity and promote microbial niche diversification^43^.

Our analysis further unveiled complementary patterns of cyanobacterial and non-cyanobacterial diazotroph richness with some overlap in richness hotspots in the Indian and Pacific Ocean (Fig. 1c and e). This result is further supported by a study of Tang and Cassar^44^ who found a high degree of niche overlap for cyanobacterial diazotrophs in those regions. Less is known about the global richness patterns of non-cyanobacterial diazotrophs^13^. Our results on increased non-cyanobacterial richness in tropical upwelling regions are consistent with findings from the Tara Oceans expedition, which identified non-cyanobacterial diazotrophs in both free-living and particle-associated fractions of nutrient-rich waters^10,45–48^. While non-cyanobacterial diazotrophs are not obligately particle-associated, their elevated richness in regions with high primary productivity and enhanced particulate organic carbon likely reflects the availability of oxygen-depleted micro environments that facilitate their activity^26^. A recent study indicated that in *Cand. Thalassolituus haligoni*, nitrate concentrations had no significant effect on nitrogen fixation, as nitrogenase activity and protein expression levels remained consistent across a range of nitrate conditions^12^.

Our results are in line with a first order control of temperature on the distribution of diazotroph species richness and lends support to the kinetic energy hypothesis, which posits that higher temperatures promote higher species diversity through increased speciation rates and the selection of warm-water-tolerant species^4,36,49,50^. Alternative hypotheses could contribute to explain the global diazotroph richness patterns. The physiological tolerance hypothesis posits that temperature limits species distribution by constraining species’ ability to tolerate extreme cold temperatures^51^, while the productivity/resources and environmental stability hypotheses highlight the role of resource availability and environmental stability in driving diversity^52^. These hypotheses are not mutually exclusive, and comparing their predictions against empirical data can lead to an understanding of the factors influencing global diazotroph richness. Other factors, such as nutrient availability, especially nitrogen, phosphorus, and iron, are critical drivers of diazotroph diversity^16^. These factors interact with temperature to create ecological conditions that support diverse diazotroph communities. Therefore, while temperature-dependent speciation provides a valuable framework for understanding species richness patterns, it should be considered in conjunction with these other environmental variables to more fully explain diazotroph diversity.

We identified an emergent positive BEF relationship between marine nitrogen fixation and diazotroph richness (Fig. 2) that remains robust across varied bin sizes (Supplementary Fig. 11), data types (Supplementary Fig. 2), and independent nitrogen fixation products (Supplementary Fig. 9). Our findings suggest that, on a macroecological scale, the covariation between diazotroph richness and nitrogen fixation reflects the manifestation of an optimal niche environment (Fig. 2; Supplementary Fig. 10). While broad-scale thermal and nutrient regimes influence both taxonomic diversity and nitrogen fixation rates, our stratification analysis suggests that this relationship is not merely a consequence of environmental covariance. The persistence of the BEF signal within narrow environmental windows suggests that richness captures unique ecological nuances, such as community-level niche filling, that are not fully resolved by abiotic predictors alone. We acknowledge that at global scales, large-scale environmental filtering might dominate observed diversity patterns^53^. However, the internal consistency of the richness-fixation link across varied environmental strata suggests that diazotroph richness is an indicator of ecosystem functioning, even when overarching physical constraints are accounted for. On local scales, dynamic processes such as mesoscale eddies or frontal zones, short-term variations in community assembly and species-specific traits may decouple nitrogen fixation from the background environmental state. Our broad-scale correlative methods preclude the identification of direct causal links, future sub-mesoscale research and high-resolution time series are required to determine how much diazotroph richness independently contributes to nitrogen fixation at the local patch scale.

We proposed that differences in life histories and resource-use strategies among diazotroph taxa promote species co-existence and optimize resource-use efficiency. This, in turn, could enhance community-level nitrogen fixation, particularly under stable environmental conditions such as those in the tropics^54^, where our SDMs project the highest normalized diazotroph richness (Fig. 1a). The high energetic costs and oxygen sensitivity of the nitrogen-fixing enzyme led to a variety of strategies enabling marine nitrogen fixation^25^. Thanks to the temporal variability in environmental conditions, several strategies may be expressed throughout the diurnal cycle, on daily or weekly scales, which may lead to a cumulative effect of sub-monthly succession patterns on total monthly and annual mean marine nitrogen fixation rates. In turn, this may lead to an emergent positive relationship between nitrogen fixation and diazotroph species richness on longer temporal scales that was then captured by our macroecological approach. A larger species pool increases the chance that certain species begin to fix marine nitrogen fixation when environmental conditions limit the growth of other species. While our results identify a clear spatial association, we hypothesize that temporal niche partitioning may be a primary mechanism explaining why regions of higher nitrogen fixation are co-located with higher richness on an annual scale. Rather than species strictly replacing one another through time, a larger species pool likely enhances the community’s aggregate functional output through niche complementarity, where different taxa occupy distinct micro-niches or utilize resources with varying efficiencies. In the relatively stable tropical environments where these peaks occur, this diversity may act as a functional buffer; while certain species may up- or down-regulate based on high-frequency environmental shifts, the continuous presence of multiple functional types ensures a higher and more stable baseline of nitrogen fixation. Consequently, we view this richness-fixation coupling as an emergent property of community assembly, where diversity facilitates a more thorough exploitation of the available environmental niche.

We also found that the loss of cyanobacterial diazotrophs from the community contributes most to the changes in community composition along the gradient of marine nitrogen fixation (Fig. 3). Rather than being driven by the selection of a single dominant taxon, the large-scale relationship emerges from the co-occurrence of multiple cyanobacterial functional groups. While we lack the species-specific abundance data required to formally test for a selection effect (where one high-performing species drives the trend), the spatial alignment of peak richness and peak fixation suggest that a diverse composition, rather than competitive dominance, is the primary feature of high-fixation regions. We find that the statistical robustness of this relationship in observational datasets is scale-dependent. While the signal is obscured at the 1° scale by local-scale stochasticity and sampling noise, a robust and statistically significant positive correlation between cyanobacterial richness and *in situ* nitrogen fixation emerges at the 20° macro-ecological scale (Supplementary Fig. 12l; Spearman’s ρ=0.27, 95% CI [0.07, 0.46]). In contrast, non-cyanobacterial richness showed a negligible relationship with *in situ* rates even when aggregated to larger scales (Supplementary Fig. 12m). However, this does not preclude the potential for richness-driven effects in the aphotic zone or deeper water columns, where the absence of cyanobacterial competition may allow non-cyanobacterial communities to exert a more detectable influence on local nitrogen cycling. This scale-emergent consistency between model projections and independent field observations suggests that the richness-fixation relationship is a robust macroecological feature that becomes detectable when data are spatiotemporally integrated. Rather than indicating a selection effect, where a few highly productive species dominate the signal, this alignment reflects the stabilization of the BEF relationship at broader spatial and temporal scales. This is consistent with observed patterns in marine plankton, where the underlying biological trends often emerge only once the high variance and noise inherent in local, discrete sampling are integrated into a larger-scale ecological context^55^.

As anthropogenic climate change continues to rise ocean temperatures and alter nutrient distributions via ocean stratification^56^, our results imply that the diversity of diazotroph richness could increase, especially in the South Pacific and the central Atlantic, as discussed in Dutkiewicz et al.^57^ and thus increase marine nitrogen fixation in the tropical and temperate ocean. While a potential increase in diazotroph richness could influence future nitrogen fixation rates, our study does not provide a budget-level analysis of nitrogen export. Therefore, whether this biotic response could significantly offset climate-driven nitrogen limitation caused by stratification remains a subject for future investigation^58^.

Our study relies on correlative models, which, while useful for identifying large-scale biodiversity-environment relationships, do not capture the underlying mechanistic processes that govern species dynamics. We acknowledge that our modeling framework captures only a limited portion of the taxonomic diversity of marine diazotrophs. To assess whether this affects our global normalized richness gradients, we systematically omitted taxa from the full set. We evaluated the stability of the resulting diversity structure using the Mean Silhouette Index. We found that the spatial structure of normalized richness remained stable (Mean Silhouette Index range: 0.43–0.45; Supplementary Fig. 4) even after removing up to 9 taxa (over 60% of the total). This indicates that the core richness patterns are largely driven by common and widespread species. As demonstrated by Jetz and Rahbak^32^ for terrestrial birds, total species richness at large scales can often be well approximated by the most frequently occurring species. To further evaluate the representativeness of our modeled richness patterns, we contrasted the patterns with independent metagenomic richness estimates from the Tara Oceans dataset^41^. We found positive correlations for total diazotroph richness (median Spearman’s ρ = 0.7, 95% CI [0.2, 0.8]; Supplementary Fig. 3a, d, g), cyanobacterial diazotroph richness (median Spearman’s ρ = 0.8, 95% CI [0.2, 0.9]; Supplementary Fig. 3b, e, h) and non-cyanobacterial diazotroph richness (median Spearman’s ρ = 0.6, 95% CI [0.2,0.8]; Supplementary Fig. 3c, f, i), supporting the conclusion that our selected taxa serve as robust proxies for large-scale biodiversity gradients despite underrepresenting total richness. While our data suggest a limited role of succession at the monthly scale (Supplementary Fig. 15), particularly in the tropical ocean, succession may play a more prominent role in temperate regions or during specific seasons. Diazotroph richness was modeled at a monthly resolution and subsequently aggregated to the annual mean scale for comparison with modeled annual nitrogen fixation rates. Although this approach facilitates large-scale comparisons, it may obscure important intra-annual variability. Supplementary Fig. 15 illustrates that diazotroph richness can vary month to month, especially in regions subject to strong seasonal forcing. Similarly, nitrogen fixation rates may undergo significant seasonal fluctuations influenced by dynamic environmental conditions such as temperature, nutrient availability, and light^8^.

Because our approach is correlative rather than mechanistic, a shared-driver explanation cannot be fully excluded and likely accounts for a portion of the richness-fixation covariance in the data. The internal consistency of the richness-fixation link across varying environmental windows, however, suggests that this relationship is not merely the outcome of environmental forcing, but represents a detectable ecological feature of the diazotroph community (Supplementary Fig. 10). We acknowledge several limitations inherent to our macroecological approach, which we aimed to address through a series of systematic investigations. While our taxonomic coverage is restricted to 15 species modeled, we found that the spatial structure of our richness patterns remained consistent (Mean Silhouette Index: 0.43–0.45; Supplementary Fig. 4) even when the species pool was reduced by 60%, and our projected gradients align with independent metagenomic richness from the Tara Oceans dataset (ρ ≥ 0.6; Supplementary Fig. 3). To investigate potential biases introduced by the integration of microscopy and sequencing data, we performed cross-datatype validations that showed spatial agreement for common taxa (ρ=0.68–0.94; Supplementary Fig. 2). Finally, while we rely on presence-absence data, the persistence of a diversity-functioning signal within narrow environmental ranges (e.g., ρ=0.51 in the 24–26°C thermal bin) and its consistency with HSI suggests that richness estimates may capture ecological nuances not fully resolved by abiotic predictors alone. Collectively, these analyses indicate that the identified global and positive BEF relationship is a persistent feature of the marine nitrogen cycle across the tested scales.

The identification of a positive global association between diazotroph richness and marine nitrogen fixation provides a foundation for deeper exploration of the ecological mechanisms driving this relationship. The occurrence of non-cyanobacterial diazotrophs in nutrient-rich upwelling regions raises further questions on their activity within such marine regions since it is not yet clear if the majority is capable of marine nitrogen fixation in the environment, although genetic elements for marine nitrogen fixation are present within their genome. The discrepancy between the genomic potential and realized functioning underscores the need for direct measurements of non-cyanobacterial specific nitrogen fixation activity. Moreover,  vigorous activity of non-cyanobacterial diazotrophs under such conditions would suggest a broader ecological niche regarding nitrogen fixation than previously recognized.

Importantly, our study’s approach of integrating disparate data sources, including microscopy and sequence-based observations allowed us to maximize the number of observations needed to train our models and to show that observations either retrieved from microscopy or sequence-based methodologies led to similar global patterns of key diazotroph taxa (Supplementary Fig. 2). This increases our confidence in merging observations from different sampling methodologies for microbial plankton taxa, which will help to enlarge the pool of observations needed for future studies that aim to constrain uncertainties related to small sampling sizes.

In summary, our results show a consistent spatial alignment between diazotroph richness and nitrogen fixation across the global ocean. This relationship suggests that the diversity of the diazotroph species pool is closely linked to the magnitude of the marine nitrogen cycle. By demonstrating that higher richness corresponds to increased functional potential, our findings indicate that positive BEF relationships exist in marine plankton, with potential implications for the regulation of global biogeochemical cycling in a changing ocean.

## Materials and methods

### Diazotroph occurrence dataset

We compiled an exhaustive dataset of diazotroph occurrences from public sources and from recent studies that focused on quantitative (counts or gene reads) and qualitative (presence-absence, non-detections) field records of planktonic diazotrophs. To unify disparate data types, we harmonized microscopy-defined species and metagenomically-derived units into comparable ecological units. Metagenomic-assigned genomes (MAGs) were assigned to molecular operational taxonomic units (mOTUs)^59,^representing species-level units, using median pairwise distances of 10 single-copy marker genes across all genomes in the mOTU online reference database^60^ and a calibrated 96.5% sequence identity cutoff^59^. By integrating morphological identifications with high-resolution genomic clusters, we captured a substantially expanded taxonomic coverage of the diazotroph community.

To minimize methodological bias introduced by combining disparate data types, specifically cell counts (microscopy), gene copy numbers (qPCR), and metagenome-based relative abundances, we converted all quantitative records to binary presence-absence data, interpreting zeros as absences. In contrast to abundances, presence-absence data tend to be a more robust and conservative choice when combining diverse datasets. Although biomass often relates to instantaneous ecosystem functioning, species richness was chosen as our primary target unit because it reflects the cumulative niche breadth and overlap across community members. According to the insurance hypothesis, the presence of a more diverse suite of species, even at low abundance, indicates a broader reservoir of metabolic potential^61^. This taxonomic diversity may support stable ecosystem processes across varying environmental conditions, with different species maintaining ecosystem functioning if environmental conditions shift. For quantitative records, a presence was defined as any observation exceeding the study-specific limit of detection,  thereby restricting richness estimates to the detectable biological component of each sample.

We compiled diazotrophic occurrence data from the Global Biodiversity Information Facility (GBIF; www.gbif.org, last access: 20 October 2021), the Ocean Biogeographic Information System (OBIS; www.obis.org/, last access: 21 October 2021), Luo et al.^62^, Tang and Cassar^44^, Gradoville et al.^22^, Detoni et al.^21^, Martinez-Gutierrez et al.^63^, Phytobase^64^, Pierella Karlusich et al.^10^ and the Ocean Microbiomics Database^41^ (Supplementary Fig. 17). We used an ocean mask^65^ to ensure that only marine taxa were included and observations displaying a doubtful taxonomic assignment in the original datasets were removed. To avoid the inclusion of outdated species names from early sampling periods, each taxon name of microscopic origin was screened against the World Register of Marine Species (WoRMS, https://www.marinespecies.org), retaining the taxa with an accepted status. Taxon names flagged as unaccepted in WoRMS were either removed or corrected by an alternative accepted name if provided in WoRMS. When the measurement method was not specified in the original datasets, we identified it from the associated original publication for each observation. Since our dataset includes both microscopy-based and sequence-based records, this step was essential for accurately categorizing and integrating the data. If no information on the measurement method was available in the original source or publication, we used the sampling date (day, month and year) as a chronological proxy to differentiate between distinct methodological eras. Samples collected prior to the 1980s were classified under microscopy-based. For some studies, the exact days were not recorded but a several-week period was given. In those cases, we assigned the specific month of collection based on the mentioned survey period (all periods < three weeks).

 Non-cyanobacterial diazotrophs were obtained from MAGs assembled by Delmont et al.^66^ and from the Ocean Microbiomics Database^41^, which compiles data from Acinas et al.^67^, Biller et al.^68^, Delmont et al.^66^, Ibarbalz et al.^9^, Klemetsen et al.^69^, Pachiadaki et al.^70^ and Sunagawa et al.^71^. The MAGs were taxonomically classified using the Genome Taxonomy Database^72^. While retaining metadata on sampling method, we discarded duplicated records across the sources based on exact IDs defined by the columns “family”, “genus”, “species”, “decimalLongitude”, “decimalLatitude”, “year”, “month”, “day”, and “depth”. 

### Open ocean environmental predictors

Environmental predictors were compiled to reflect key dimensions of microbial plankton niches that shape species’ distributions via effects on physiology, growth or species competition^36,73,74^. Since we focused on the diazotroph community of the global offshore surface ocean (samples within the surface mixed-layer), we reduced the confounding influences of complex and fertile coastal environments by excluding data from seas shallower than 200 m^75^ and from regions characterized by climatological surface salinities below 20^76^.

### Species distribution models

SDMs fit statistical associations between species’ observed occurrences and environmental variables; i.e., they estimate a species’ realized ecological niche by fitting a response curve between the distributions of occurrence data and environmental covariates (Supplementary Fig. 18a)^74^. SDMs provide a useful framework to explore large-scale distributions of microbial plankton species. They assume that: (i) species are not dispersal-limited in the open ocean^77^; (ii) species are primarily controlled by abiotic environmental factors in their global distribution^77^, and rapidly respond when conditions turn suitable^78^. Because the distribution of diazotrophic taxa varies seasonally^8^, we matched each occurrence record to the corresponding monthly climatological values of the environmental predictors (e.g., observations in March were paired with March temperature). This ensured that SDMs were trained using environmental conditions that reflect the seasonal context in which the observations were made. An overview of the number of samples for each season and latitude is visualized in Supplementary Fig. 17d. Each taxon was modeled using these temporally matched datasets, and the resulting SDMs were then projected onto monthly global environmental fields at a 1° x 1° resolution to generate species-specific HSIs for each month of the year. Binary presence-absence maps were then obtained by applying a probability threshold to the monthly HSI maps. We follow the standard SDM ensemble framework of Righetti et al.^36^ and Benedetti et al.^35^ which has been shown to robustly model species distributions and the associated emergent patterns of species diversity. We further developed an ensemble of SDMs that address three key sources of uncertainty: (i) sampling bias, (ii) predictor choice, and (iii) algorithm choice.

We binned the species’ presences and zero-abundance records into the monthly 1° × 1° cell grid to match the resolution of the environmental predictors. Multiple observations per species and 1° cell that came from the same month although from potentially different years were counted as a single monthly record. To capture a broad range of diazotroph taxa, we retained species with at least 22 presence records for subsequent SDM analysis. 

### Target-group approaches to sample background data

To inform the correlative SDMs about the parts of the environmental space that are less suitable for the species to be present, we had to generate background data (also termed “pseudo-absences” in the literature). We selected environmental background data for each species, using the target-group approach^36,81^. This served two purposes: (i) ensure that background sampling followed a sampling scheme similar to that of the presence data, thereby balancing presence data distribution and sampling bias when fitting SDMs; (ii) ensure that extensive ocean areas characterized by low or missing sampling efforts were not misclassified as areas of low species’ habitat suitability. Background selection strategies were applied consistently across modeling algorithms (GAM, GLM, and RF), with adjustments only in the ratio of presence to background points to match algorithm-specific requirements. We use a larger number of pseudo-absences (presence/absence ratio = 1/10) with equal weighting for regression-based techniques and a ratio of 1/1 for tree-based techniques as suggested by Barbet‐Massin et al. (2012)^82^.

We defined three different target groups: the “total target group”, the “group-specific target group” and the “cruise-specific target group”. The “total target group” approach included taxonomic records from Phytobase^64^ which fell into the surface ocean mixed-layer, excluding records from the comparably larger-sized diatoms and dinoflagellates. The “group-specific target group” consisted of all locations from the compiled diazotroph database and the “cruise-specific target group” provided background information from cruises that used the same sampling method. This use of varying background selection strategies is a powerful tool if the particular method is applied in a sufficiently broad environmental context and across multiple taxa. In the context of mostly data-deficient diazotrophs, several methods have only been applied in certain ocean basins and without a regular grid. Therefore, to provide enough environmental variability for the modeling it is important to strive for extensive datasets, merging observations that originate from varying sampling methodologies.

Under all three configurations of target groups, we sampled the background data in a stratified manner from the target group following the procedure of Righetti et al.^36^, summarized hereafter. We incorporated two environmental gradients (sea surface temperature and mixed layer depth) during the background selection to ensure that the breadth of these key environmental factors was reflected in the background of each taxon. Background data were both sampled with overlapping option^81^, meaning that the taxon modeled remained part of the sampling density estimation and could provide background data itself, and with non-overlapping option, referring to the case where the presence cells of the taxon modeled were excluded from the background. While the overlapping option generates a background that is more general (i.e., reflecting the environmental conditions sampled in the study domain) the latter is more specific (i.e., creating pseudo-absences).

In marine systems, dispersal limitation is generally weak for planktonic microbes, and large-scale distributions are shaped primarily by environmental conditions rather than geographic isolation^55,77,83^. Thus, our target-group background approaches capture both sampling effort and broad environmental gradients (Supplementary Fig. 20).

### Algorithm and complexity choice

Statistical algorithm and complexity choice represent main sources of uncertainty in studies relying on SDMs. We constructed SDMs based on either General Linear Models (GLMs; using the R package “stats”), General Additive Models (GAMs; R package “mgcv”), or Random Forests (RFs; R package “randomForest”), as three algorithms of increasing statistical response shape complexity. We used a relatively small number of predictors (n=3–4) and fitted simple response shapes to account for variation in sample size across species. Taxa with 22 to 36 presences were modeled using three environmental predictors per SDM, corresponding to a minimum ratio of approximately seven observations per predictor for the smaller sample size group, and at least nine observations per predictor for the larger group, both of which meet or exceed commonly accepted lower thresholds for SDM parameterization (Supplementary Fig. 19)^36,74,79^. GAM used smoothing terms with four basis dimensions (k=4), estimated by penalized regression splines without penalization to zero for single variables. To balance the overall weight of presences versus background data per species, background data in GAM and GLM were weighted by the ratio of species’ presence to background data points. RFs included 4’000 trees, simple terms, and single-end node size. The weighting of data in individual RF trees was balanced by randomly subsampling the same amounts of background data as the species had presences as suggested by Barbet‐Massin et al.^82^. In cases where the sampling of absences resulted in a lower number than presences due to the lack of potential locations valid for drawing absences, presences have been downsampled to the number of absences found, to keep the 1:1 ratio, when running the RF.

### Predictor ranking and selection for member models.

In addition to algorithm choice, predictor choice represents a potentially important source of uncertainty in the present SDMs, as the environmental variables controlling the spatial distributions of planktonic diazotrophs remain poorly known. We fitted single-factor GLM, GAM, and RF models to the presence versus background data of each taxon, for each candidate predictor following Righetti et al. 2019^36^. Model explanatory skill was evaluated using the adjusted *D*^2^ for GLM, adjusted *R*^2^ for GAM and the Out-Of-Bag Error statistic for RF. For each species, predictors were ranked according to these statistics, and a predictor ensemble using the mean variable ranks was obtained across GLM, GAM, and RF, which served as a basis for predictor selection.

To capture predictor-based uncertainty in SDMs we prepared five sets of four predictors, used under the various model configurations and three algorithms employed SDMs. We used a randomization approach to select the four predictors per member model, using the predictor pre-ranking of each taxon as a basis, to catch a broad environmental spectrum when modeling each taxon (Supplementary Fig. 18c). We computed pairwise Spearman’s rank correlation coefficients between all predictors across the study area, omitting correlations between predictors > |0.7| in individual SDMs, since predictor collinearity can inflate the standard errors of regression model parameters and inflate their variance, leading to biased projections^85^.

### Evaluation of member models and ensemble prediction.

For each species, we evaluated the predictive skill of each SDM ensemble member based on random fourfold cross-validation. In this cross-validation, the species’ presences and background data were randomly split into four fractions with approximately equal data quantities. The ensemble member was iteratively trained on 75% of the data and the predictions were evaluated against the remaining 25%. We used the True Skill Statistic (TSS) to quantify model skill (i.e., for each member model, per taxon). The TSS ranges from -1 to +1 with values greater than zero indicating models performing better than random. We retained members showing a TSS score of at least 0.3 to build the SDM ensemble framework. Supplementary Fig. 1 shows a heatmap of TSS scores of each successfully modeled diazotrophs across all 90 member SDMs. Successful member SDMs were projected globally onto monthly (n=12 months) environmental data fields, yielding species-level maps of HSI. Before estimating species richness, we converted the HSI maps to presence-absence maps based on the probability threshold that maximizes the TSS using the function “optimal.thresholds” from the PresenceAbsence package in R^86^.

### Diazotroph diversity

Richness is the simplest measure of diversity and corresponds to the number of species present within an assemblage (i.e., grid cell). We derived monthly presence-absence maps from the up to 90 successful member SDMs (TSS ≥ 0.3) for each species. The member SDMs were aggregated into 90 species richness ensemble members at 1° spatial and monthly (n = 12) resolution. In each particular ensemble member, the corresponding successful presence-absence member SDMs were stacked and divided by the number of species successfully modeled, resulting in monthly normalized richness estimates. We derived mean annual normalized richness estimates presented in the main maps by averaging the twelve monthly normalized richness values. To assess the robustness of the final estimate to SDM member thresholding we additionally generated a HSI based richness metric (summing probabilities without thresholding and normalizing by the number of species) and we found strong agreement within all diazotroph groups (Spearman’s ρ ≥ 0.95, 95% CI [>0.93, 1]) between the HSI and presence-absence based estimates (Supplementary Fig. 21). The final map on global annual normalized diazotroph richness distribution is thus computed based on a 90-member ensemble framework spanning three different SDMs (GLM, GAM, RF), six different background selection strategies (total target-group, group-specific, cruise-specific with overlapping and non-overlapping options) and five predictor settings for each out of 15 species. This framework covers the main sources of uncertainty reported in recent SDM based studies and increases the generalization of the main diversity patterns we estimate^87,88^.

To maximize use of available data, we modeled taxa at the genus level when species-level data were strongly limited (e.g., *Trichodesmium*), while maintaining separate models at species-level (e.g., *T. erythraeum*, *T. thiebautii*). Species-level records were aggregated to genus-level records, after discarding species identity and removing duplicated records. 

To estimate beta diversity and investigate the ecological processes underlying the global gradient of diazotroph species richness, we calculated the Jaccard index and its two components: (i) species turnover and (ii) species nestedness. Since these metrics require consistent community composition data over time, we first derived annual species presence maps for each ensemble member. A taxon was considered present in a given grid cell for the year if it was detected in at least one of the twelve monthly presence-absence maps. Thus, a value of 1 indicates an annual presence of all diazotrophs at the specified locations for at least one month annually. Using these annual presence maps, we computed pairwise dissimilarities and decomposed beta diversity into turnover and nestedness components for each ensemble member. Finally, we averaged the results across all ensemble members to obtain ensemble mean estimates of beta diversity and its components. We used the *beta.pair* function from the *betapart* package in R^40^ according to the equation:

1 $${ }_{jac} ={ }_{jtu} + { }_{jne} = \frac{b + c}{a + b + c} = \frac{2b}{2b + a} + (\frac{c - b}{a + b + c}) (\frac{a}{2b + a})$$

where ${ }_{jac}$ is the Jaccard dissimilarity, ${ }_{jtu}$ the turnover component of Jaccard dissimilarity, and ${ }_{jne}$ the nestedness component of Jaccard dissimilarity. We calculate the pairwise dissimilarity between all site pairs and then average the results for each grid cell across all other grid cells. In this calculation, ‘*a*’ represents the number of shared species between two cells, ‘*b*’ stands for the number of species unique to the poorer site, and ‘*c*’ denotes the number of species unique to the richer site. All beta diversity components are based on the annual presence maps (as defined above). Moreover, the beta ratio was computed as ${ }_{ratio} ={ }_{jne}/{ }_{jac}$ to identify which component has the highest contribution to total dissimilarity. Here, a ${ }_{ratio}$ that is higher than 0.5 would therefore indicate that the region is dominated by nestedness rather than species turnover and alternatively a value lower than 0.5 would indicate the opposite.

### Nitrogen fixation rates

To analyze the correlation between global marine biological nitrogen fixation and diazotroph richness, we used the global marine nitrogen fixation estimates originating from Wang et al.^24^ (Supplementary Fig. 8a) and Tang et al.^37^ (Supplementary Fig. 8 b-c). Wang et al.^24^ published global marine nitrogen fixation rates from the Community Earth System Model (CESM) model simulations (https://www.cesm.ucar.edu/models), where nitrogen cycle simulations were conducted using a modified version of the ocean component. Tang et al.^37^ employ machine learning algorithms (Random Forest and Support Vector Regression) that use environmental predictors to estimate global marine biological nitrogen fixation.

Additionally, global *in situ* nitrogen fixation rates were compiled from three independent observational databases and studies^8,38,39^. Initial spatial standardizations were performed to project all sample locations onto a continuous -180° to 180° longitude and -90° to 90° latitude framework. The measurements provided by Shao et al.^8^ and Chowdhury et al.^38^ were reported as vertically integrated daily rates (μmol N m⁻^2^ d⁻^1^), whereas the Raes et al.^39^ dataset provided volumetric daily rates (nmol N L⁻^1^ d⁻^1^).

To prevent highly localized, extreme biological events such as *Trichodesmium* blooms from disproportionately biasing the results, a data-driven outlier exclusion protocol was implemented. Because the datasets were collected using different methodologies and reported in different units, outlier detection was executed on each dataset independently prior to unit harmonization. For each dataset, strictly positive, finite measurements were isolated, and an upper boundary was established at the 95^th^ percentile of the respective distribution. Observations exceeding this dataset-specific 95^th^ percentile threshold were excluded. This outlier removal ensures that the resulting unified dataset reflects a conservative and statistically representative baseline of global diazotrophic activity.

After removing outliers, the datasets were harmonized to a common metric of daily vertically integrated nitrogen fixation (μmol N m⁻^2^ d⁻^1^). Because the Raes et al.^39^ dataset reported volumetric surface rates (nmol N L⁻^1^ d⁻^1^), these values were integrated over a biologically relevant depth proxy. The integration depth for each volumetric sample was defined dynamically as the shallower of the local Mixed Layer Depth (MLD) or the Euphotic Zone Depth (Zeu), with Zeu estimated based on the depth of the 1% surface photosynthetic active radiation threshold. Multiplying the volumetric rate by this dynamically assigned proxy depth yielded integrated daily rates directly comparable to the Shao and Chowdhury datasets.

To reach comparison with model projections and avoid biases inherent in annualizing daily snapshots, we implemented a temporally matched monthly aggregation. Daily *in situ* rates were binned by month and grid cell, and we computed monthly means and medians for each grid cell. To capture a representative local monthly state and further mitigate the impact of stochastic single-day events, we filtered the dataset to include only grid cells with at least two independent observations. These monthly aggregated field observations were then directly paired with the corresponding monthly model outputs for each richness ensemble member. To account for the noise-to-signal ratio of sparse observational data at fine resolutions, this matching procedure was executed across a range of spatial scales, aggregating the gridded observations and model outputs into 1°, 5°, 10°, and 20° boxes. This multi-scale approach allows for the identification of emergent biological signals as they transition from local-scale stochasticity to macro-ecological spatial dependence.

To characterize the transition in diazotroph community structure across varying levels of ecosystem function, we categorized global grid cells into deciles (10^th^ to 90^th^ percentiles) based on their annual nitrogen fixation rates (using the Wang et al.^24^ product as a reference). For each percentile bin, we aggregated the annual ensemble mean HSI for the 15 key diazotroph taxa, including cyanobacterial and non-cyanobacterial taxa. We then calculated the relative contribution of each taxon’s HSI to the total community suitability within each decile. This approach allows for a spatially independent assessment of how taxonomic dominance and community assembly shift along a global gradient of nitrogen fixation intensity, moving from low-flux oligotrophic regions to high-flux hotspots.

#### Uncertainty analysis

We assessed the influence of the choices made in the SDM framework on our final estimate of mean annual normalized diazotroph species richness. Specifically, we evaluated uncertainty stemming from three main sources: background selection, predictor selection, and algorithm choice. Normalized species richness was estimated using all resulting SDM outputs, and the CV was used to quantify spatial variability in the predicted global richness across models in addition to global maps showing the standard deviation across ensemble members (Supplementary Fig. 22).

To account for uncertainties associated with differences in sampling methodologies, we partitioned the full occurrence dataset into microscopy-based and sequence-based observations. For the subset of taxa detected by both methods (*Richelia intracellularis*, *Trichodesmium thiebautii*, and *Trichodesmium erythraeum*), we independently reran the species distribution modeling pipeline using each data type. This allowed us to assess the consistency of SDM outputs across observation methods (Supplementary Fig. 2). To assess the effect of removing species on clustering quality on the global richness projection, we implemented a function to calculate the Silhouette Index^89^ (Supplementary Fig. 4), using hierarchical clustering. We performed a permutation test to calculate the Mean Silhouette Index after sequentially removing 1 to 14 species from the dataset. For each number of removed species, 100 iterations were conducted to ensure robustness, with the mean Silhouette Index recorded for each removal scenario.

To further assess the robustness of our global diversity pattern, we used the Ocean Microbiomics Database^41^ that consists of 34,815 genomes from 6 different metagenomic studies. Using this genome collection, KEGG annotations were retrieved for each protein set per genome. An organism was considered as a diazotrophic microbe if all three genes, nifH (K02588), nifD (K02586), and nifK (K02591), were present. Within-sample richness was calculated for each sample, and Spearman correlations were computed to compare these observations with our model-derived diversity patterns for total, cyanobacterial, and non-cyanobacterial diazotroph diversity.

To robustly evaluate the relationships between diazotroph richness and nitrogen fixation rates while accounting for spatial autocorrelation, which can lead to overinflated significance in global datasets, we implemented a two-stage spatial analysis framework involving empirical variogram estimation and spatial block bootstrapping.

We first determined the spatial decorrelation range of the relationship between richness and nitrogen fixation to inform the appropriate scale for statistical independence. Because the units and scales of the various nitrogen fixation products^24,37^ differed, we extracted spatial residuals by fitting a GAM ( N_fixation​_∼s(Richness) ) using the *mgcv* package in R. This model utilized Thin Plate Regression Splines as the default smoothing basis, with the basis dimension (k) automatically optimized via Restricted Maximum Likelihood to ensure that the residuals captured the spatial variance not explained by the primary richness effect.

For each dataset, we computed an empirical semi-variogram on a randomized subsample of 5,000 grid cells to reduce computational efforts. Semi-variance was calculated across a distance of 0° to 80° using 41 lag bins. We then fitted a theoretical spherical model to these empirical variograms using the *geoR* package to estimate the effective range, the distance beyond which spatial residuals are no longer significantly correlated (Supplementary Fig. 23).

The empirically derived ranges were used to define the dimensions of spatial blocks for a block bootstrap procedure. While spatial autocorrelation in coarse 1°×1° climatological products can mathematically extend across large distances, these ranges often exceed the biologically relevant scales of plankton patchiness. Consequently, we capped the spatial blocks at a maximum of 20°^90^ and further tested smaller scales of 5° and 10° to ensure robustness.

For both the global 1° grid and the matched nitrogen fixation datasets utilized in our study, we performed 1’000 bootstrap iterations. In each iteration, spatial blocks were sampled with replacement, and all data points falling within the selected blocks were aggregated to form a bootstrap resample. We then calculated Spearman’s rank correlation (ρ), Mean Absolute Error (MAE), and Mean Error (ME) for each iteration. We report the median and the 95% confidence intervals (derived from the 2.5^th^ and 97.5^th^ percentiles of the bootstrap distribution) for all correlations computed in this study. A relationship was considered statistically robust if the 95% confidence interval for Spearman’s ρ did not overlap with zero.

To evaluate whether the observed positive relationship between diazotroph richness and nitrogen fixation represents an independent biological signal or a secondary effect of shared environmental drivers, we conducted an environmental stratification analysis. This approach tests the 'shared-driver' hypothesis by partitioning the global 1°×1° grid cells into discrete environmental windows where the variance of primary abiotic drivers is minimized. Specifically, we binned the data into 2°C sea surface temperature increments (20–22,22–24,24–26, and 26–28°C) and five discrete net primary production strata. Within each stratum, we calculated the Spearman rank correlation (ρ) between nitrogen fixation and each of the 90 ensemble members. The core assumption of this test is that if global correlations were purely a byproduct of a shared response to broad thermal or productivity gradients, the relationship should become non-significant when those gradients are restricted. We performed a sensitivity analysis across varying bin sizes (sea surface temperature: 1°C to 4°C; net primary production: 100 to 400 mg C m^−2^ d^−1^), which yielded consistent results across all test resolutions.

## Supplementary Information

Supplementary Information.

## Data Availability

Data used for this analysis are publicly available. The dataset is stored at the ETH Zurich Research Collection(10.3929/ethz-b-000635803). All codes are publicly available at the GitHub repository at https://github.com/Domle/Notion.git.
