# Supplementary material for: Nitrogen fixation rates increase with diazotroph richness in the global ocean
Source: Sci Rep. 2026 Jul 11;16:25295. doi: 10.1038/s41598-026-61132-2 (PMC13473530; doi:10.1038/s41598-026-61132-2)
Supplement: Supplementary file 1 [file 41598_2026_61132_MOESM1_ESM.pdf]

Supplementary References for

Nitrogen fixation rates increase with diazotroph richness in the global ocean

Dominic Eriksson*, Damiano Righetti, Fabio Benedetti, Nicolas Gruber, Lucas Paoli, Guillem Salazar,

Shinichi Sunagawa*, Meike Vogt*

*Corresponding author. Email:deriksson@ethz.ch, ssunagawa@ethz.ch, meike.vogt@env.ethz.ch

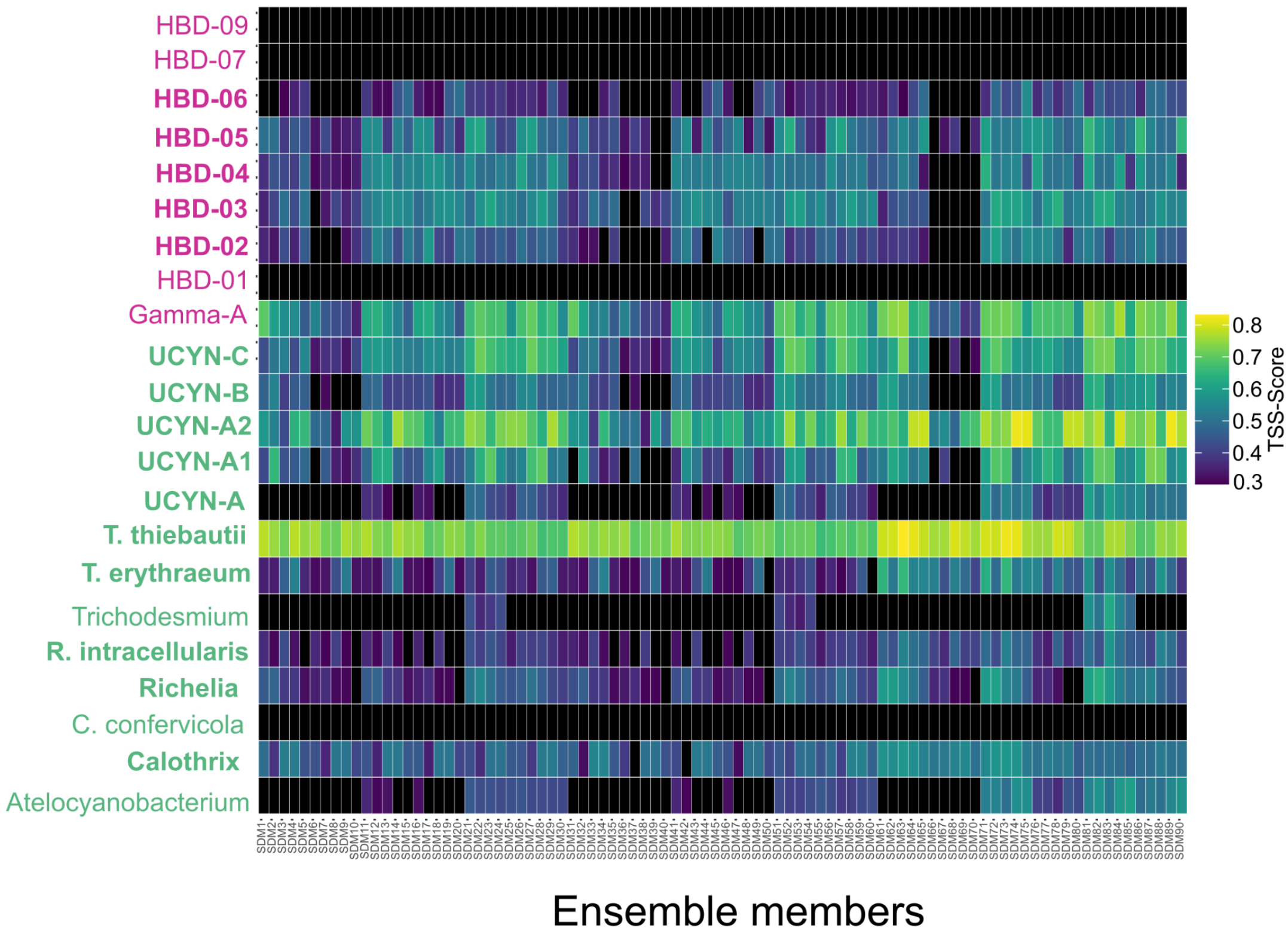

Supplementary Figure 1. Model performance on diazotroph distributions. The heatmap illustrates the performance of species distribution models for diazotrophs in the surface ocean (samples within the mixed layer depth), based on the True Skill Statistic scores (TSS). The heatmap combines both cyanobacterial (green) and non-cyanobacterial (purple) diazotrophs. Each row represents a taxon, while columns correspond to individual ensemble model members. TSS scores below 0.3 were marked black and only taxa with a TSS score > 0.3 in at least two-thirds of the ensemble members were considered in the analysis. Those taxa are marked in bold.

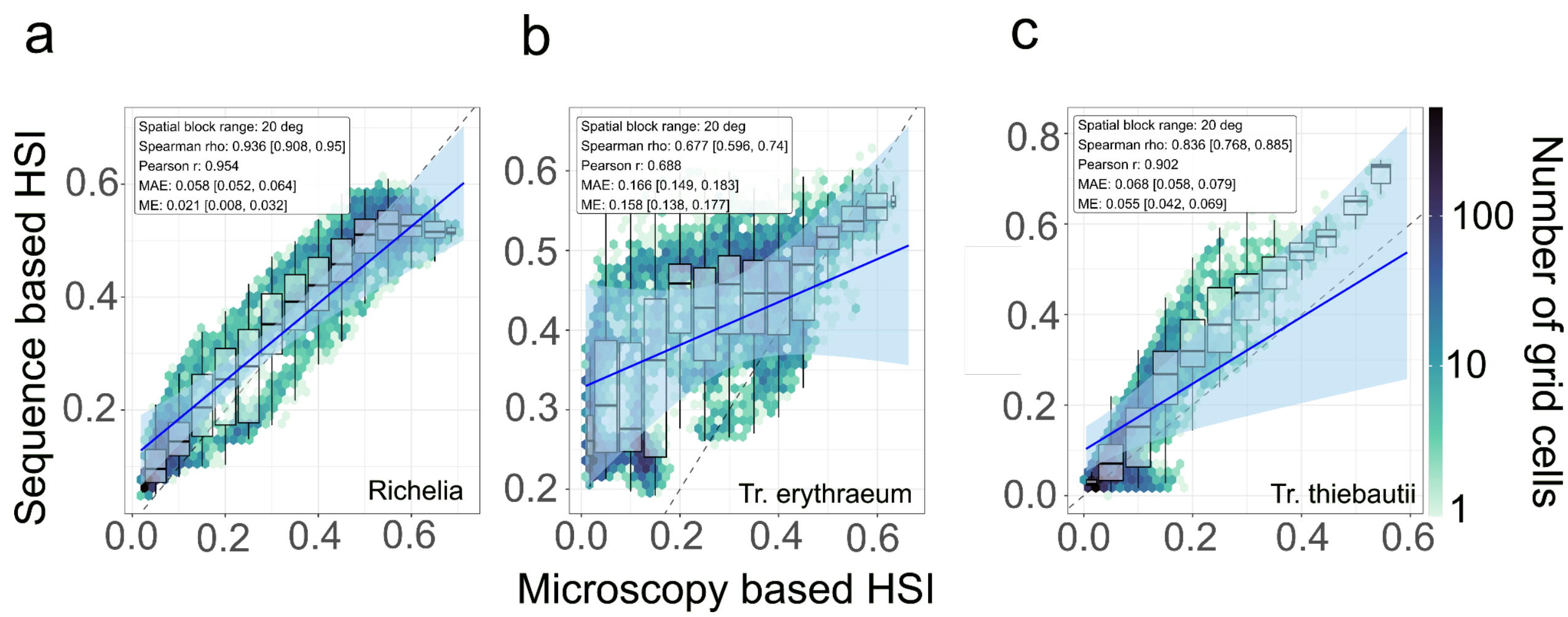

Supplementary Figure 2. Spatial relationships between microscopy-based and sequence-based annual habitat suitability predictions for overlapping diazotroph taxa. Panels show taxon-wise (a: *Richelia*; b: *Trichodesmium erythraeum*, c: *Trichodesmium thiebautii*) paired values extracted from identical global 1x1 degree grid cells. Hexagons represent the density of paired observation. Overlaid boxplots summarize the distribution of sequence-based habitat suitability within fixed microscopy-based richness bins (width = 0.05). The blue line and shaded ribbon indicate the ensemble mean linear fit and +/- 1 standard deviation across richness realization pairings (n = 90). Insets report spatial block bootstrap diagnostics based on taxon specific variogram informed block sizes: Spearman's rank correlation (rho), mean absolute error (MAE), and mean error (ME), each shown as the median with 95% confidence intervals from 1,000 bootstrap iterations.

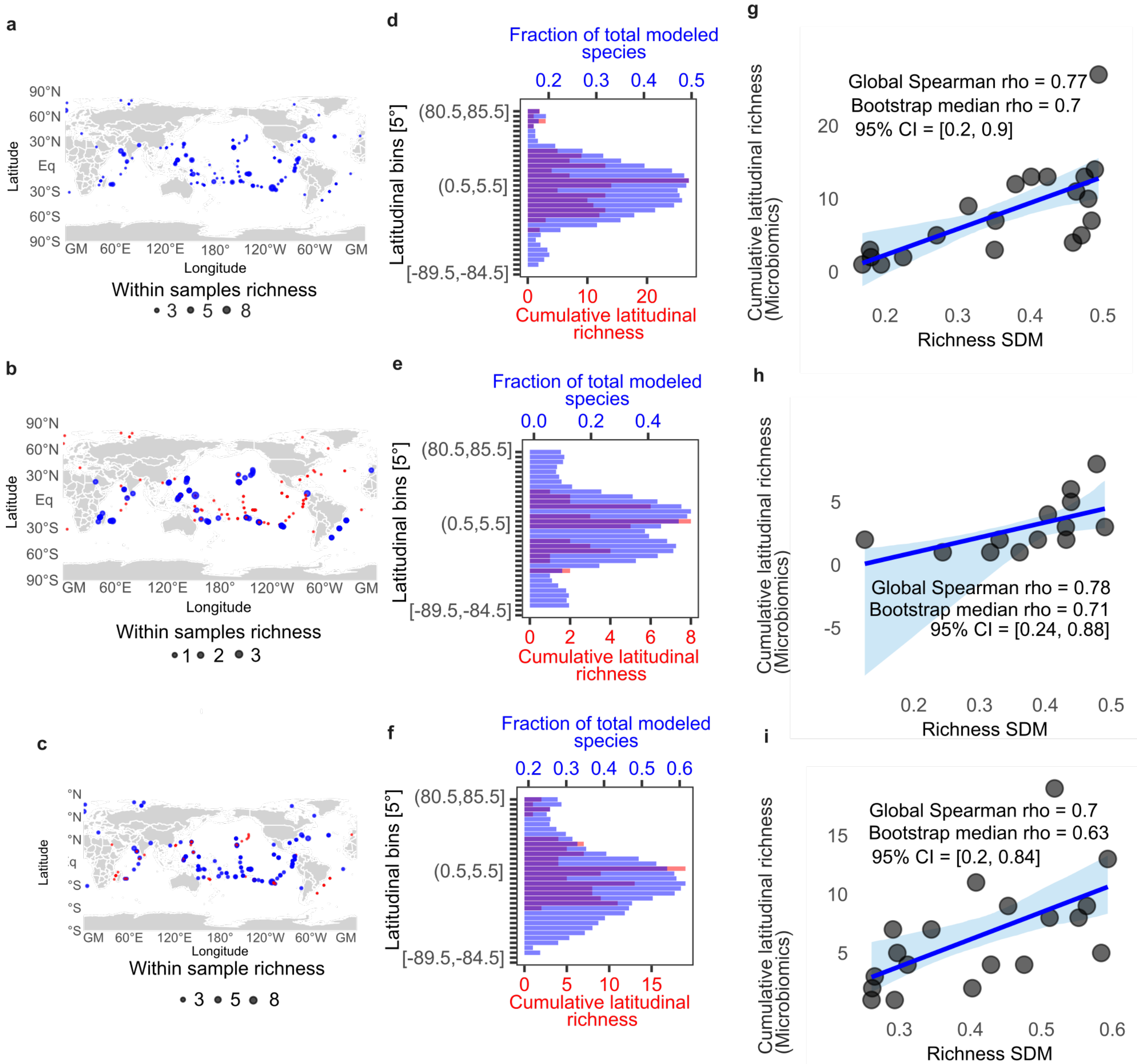

Supplementary Figure 3. Relationship between within-sample richness and modeled richness. This figure presents the global and latitudinal distribution of diazotroph richness in ocean surface waters (0–200 m depth) based on the Ocean Microbiomics Database[1] and species distribution modeling (SDM) based projections, focusing on total diazotrophs, cyanobacterial diazotrophs, and non-cyanobacterial diazotrophs. Each panel includes a world map depicting sample locations from the Ocean Microbiomics Database. **a-c**, with points scaled by within-sample richness and color-coded to indicate the presence (blue) or

absence (red) of diazotrophs. Adjacent to the maps, latitudinal gradients illustrate cumulative richness across 5° latitude bins, with observed richness from the Ocean Microbiomics Database shown in red and the fraction of total diazotrophs predicted by SDM (total number of modeled diazotrophs = 15) shown in blue **d-f**. The number of samples per bin, derived from the Ocean Microbiomics Database, is annotated. Finally, each panel includes a scatterplot correlating modeled richness (SDM) with observed richness (Ocean Microbiomics Database), displaying Spearman's correlation coefficient (ρ) and a bootstrap-based 95% uncertainty ribbon derived from spatial block resampling of adjacent latitude bins. Global, bootstrapped median correlations are annotated within each scatter plot alongside the 95% confidence intervals.

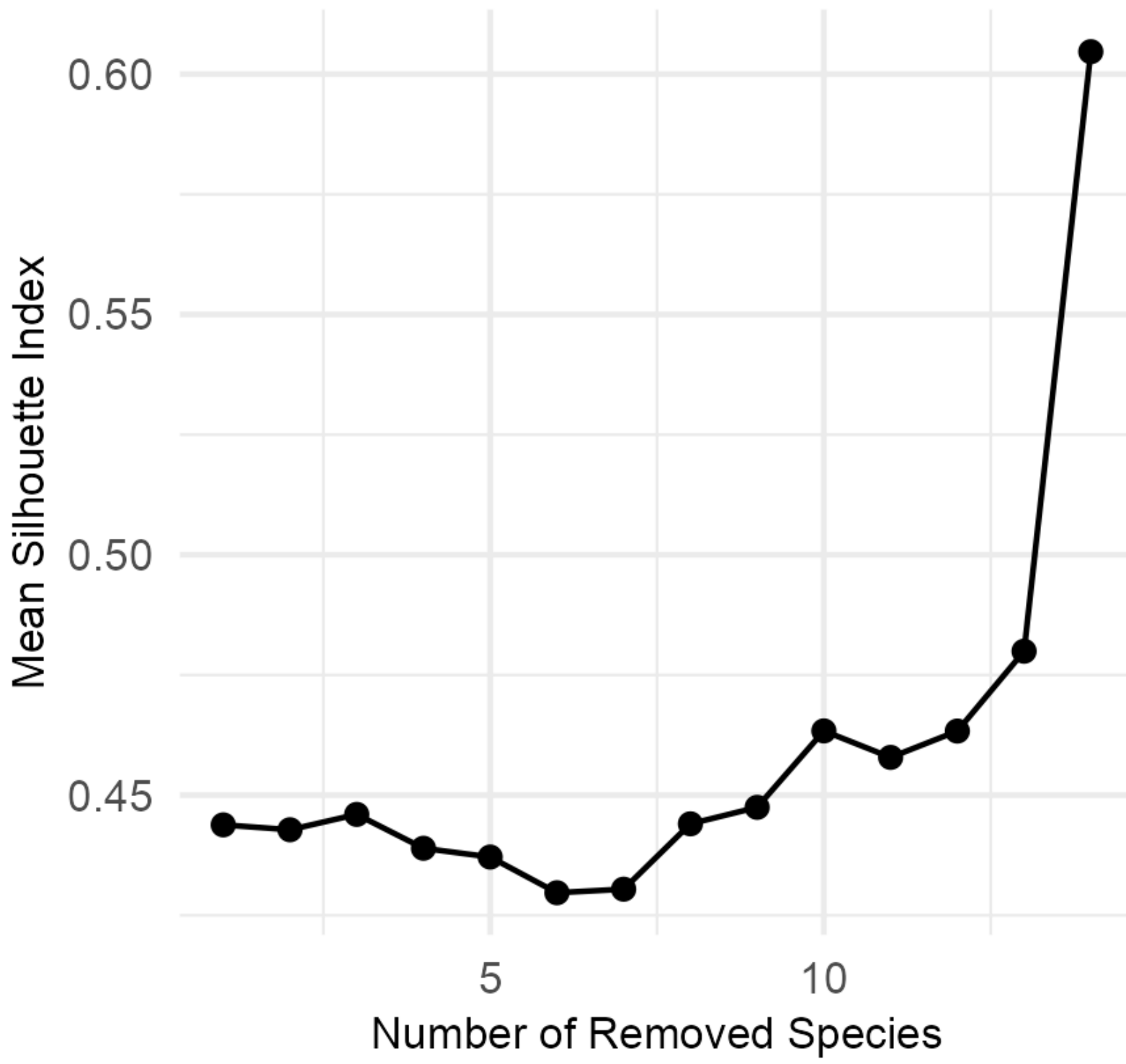

Supplementary Figure 4. Impact of species removal on richness estimation. The plot depicts the Mean Silhouette Index calculated after sequentially removing 1 to 14 species from the dataset. The x-axis represents the number of species removed, ranging from 1 to 14, and the y-axis indicates the corresponding Mean Silhouette Index values.

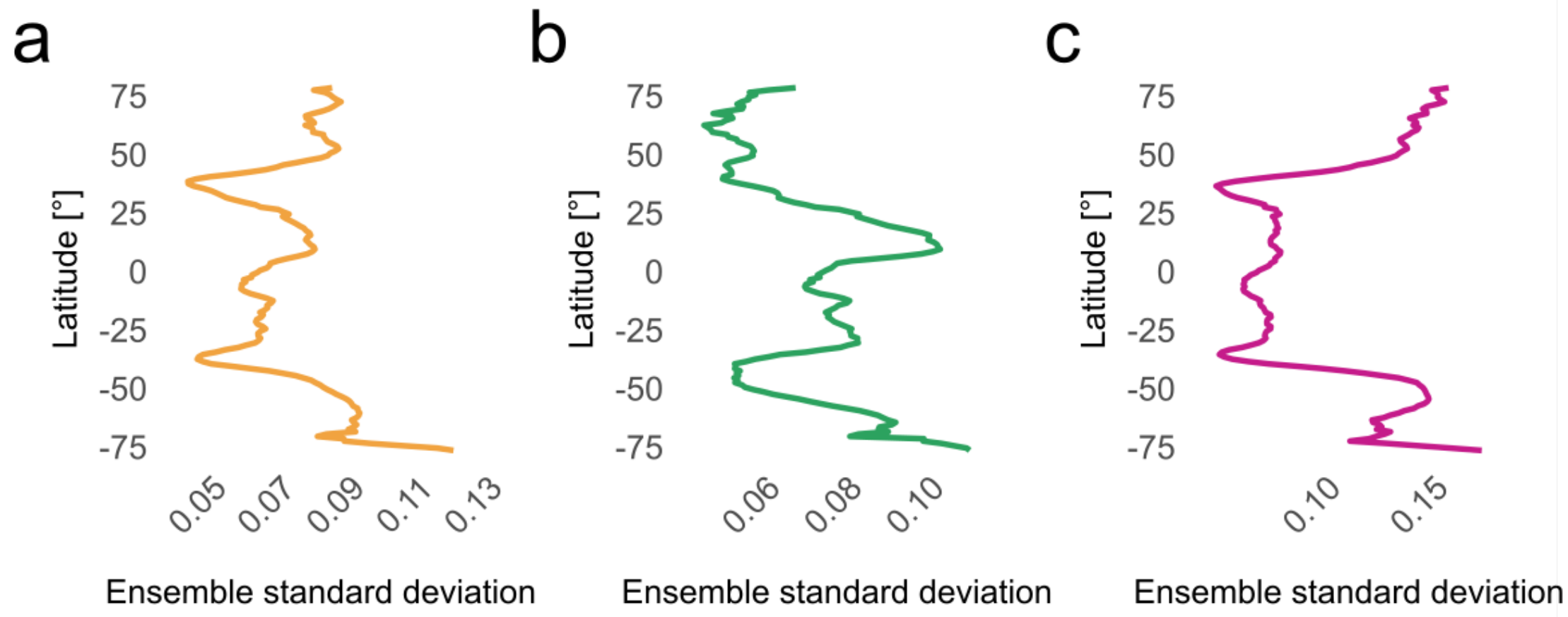

Supplementary Figure 5. Standard deviations of latitudinal richness across richness realizations. Standard deviations of latitudinal richness gradients across richness realizations for **a** total, **b** cyanobacterial, and **c** non-cyanobacterial diazotroph communities. Latitudes are binned into 1° (y-axis) and x-axis represents ensemble standard deviation.

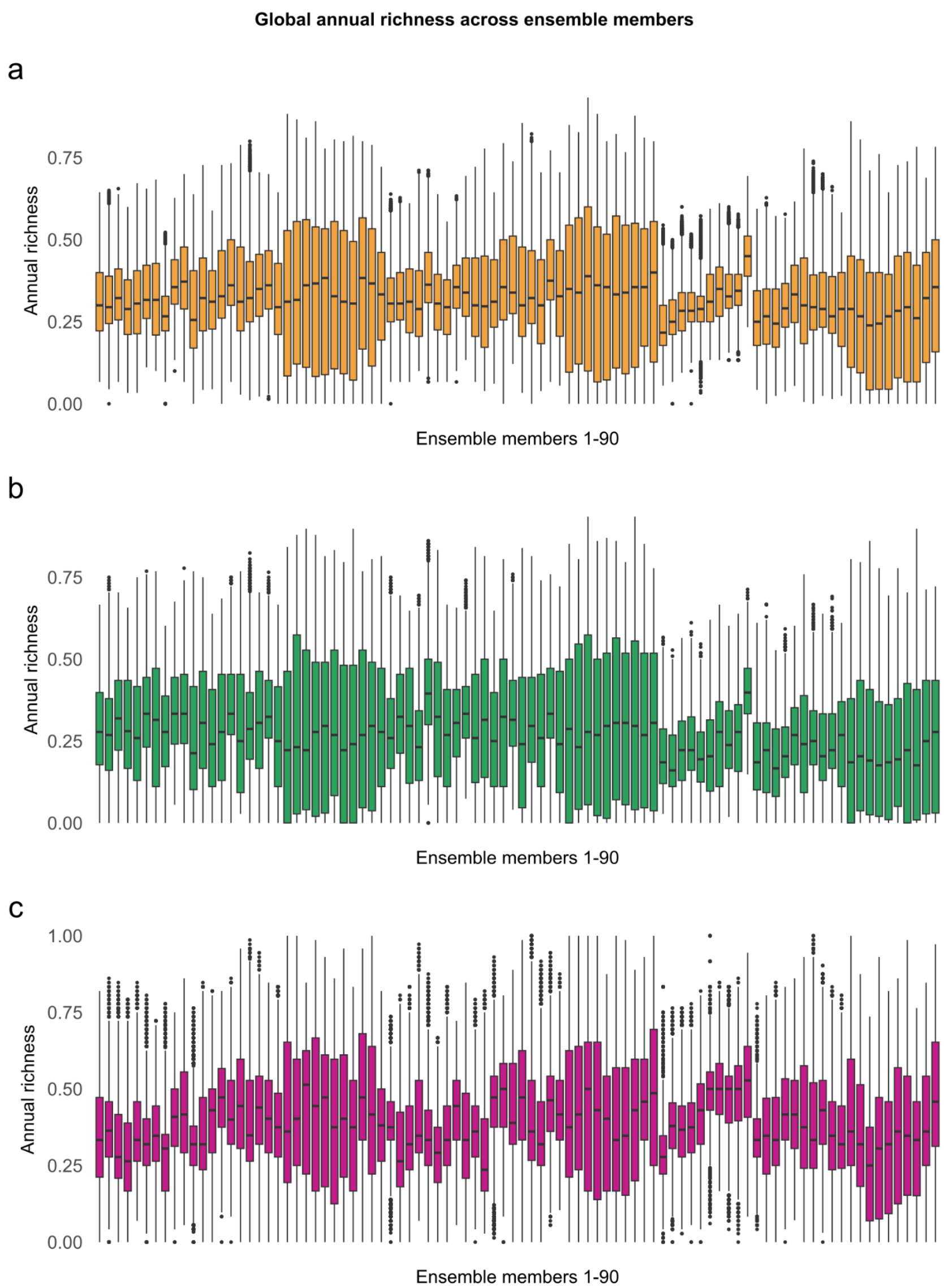

Supplementary Figure 6. Global annual diazotroph richness across richness realizations. Boxplots showing the range of annual diazotroph richness estimates (y-axis) across each one of the 90 richness realizations (x-axis) for **a** total (n = 15), **b** cyanobacterial (n = 9), and **c** non-cyanobacterial (n = 6) diazotroph communities. The annual diazotroph richness is computed with each grid cell covering 1° longitude by 1° latitude and has been normalized by the number of taxa. An annual presence has been assigned when a taxon was present at least once in twelve months.

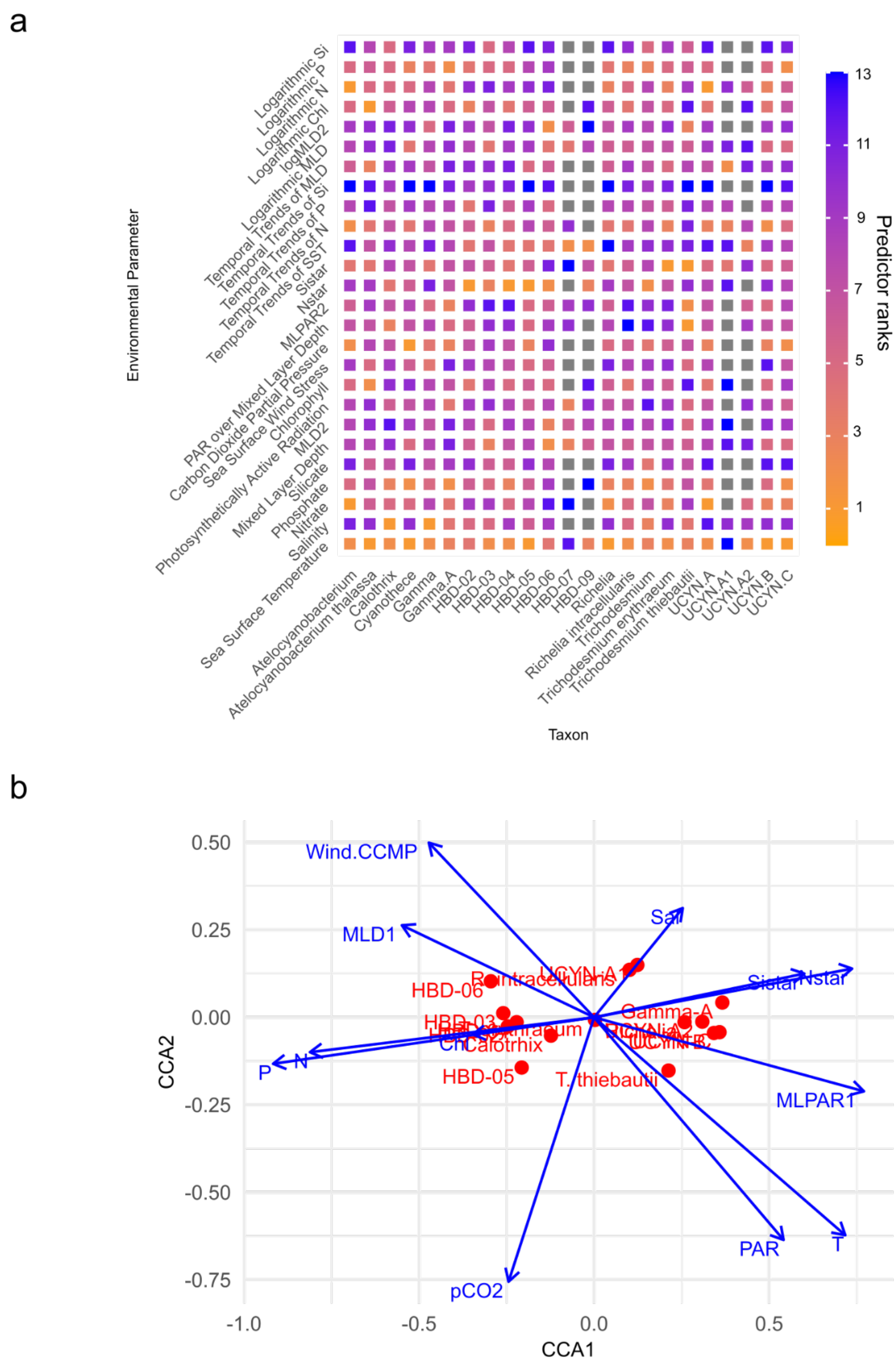

Supplementary Figure 7. Environmental drivers of diazotroph distribution. a Mean ranking of environmental parameters based on single factor analysis on each diazotroph taxon applying three different algorithms namely General Linear Model, General Additive Model, and a Random Forest. Environmental variables: T (sea surface temperature, °C), Sal (sea surface salinity), N (nitrate, μM), P (phosphate, μM), Si (silicic acid, μM), MLD1 (mixed layer depth, meters), PAR (photosynthetically active radiation, μmol m^{-2} s^{-1}), Chl

(chlorophyll, μg l^{-1}), Wind.CCMP (sea surface wind stress, m s^{-1}), pCO2 (carbon dioxide partial pressure in the surface sea, μatm), MLPAR1 (photosynthetically available radiation over the mixed layer depth, μmol $m^{-2}s^{-1}$), Nstar (excess concentration of nitrate in relation to the Redfield ratio, μM), Sistar (the ratio of nitrate to silicic acid, μM), dT_dt (temporal trends of sea surface temperature, °C), dN_dt (temporal trends of nitrate, μM), dMLD1_dt (temporal trends of mixed layer depth, meters), logMLD1 (logarithmic mixed layer depth, meters), logChl (logarithmic chlorophyll concentration, μg l^{-1}), logN (logarithmic nitrate concentration, μM), logP (logarithmic phosphate concentration, μM), logSi (logarithmic silicic acid concentration, μM). **b** Non-metric multidimensional scaling for the main environmental predictors ranked highest. Centroids have been computed on the annually averaged projected habitat suitability indices projected by our SDM on a 1° longitude by 1° latitude spatial resolution.

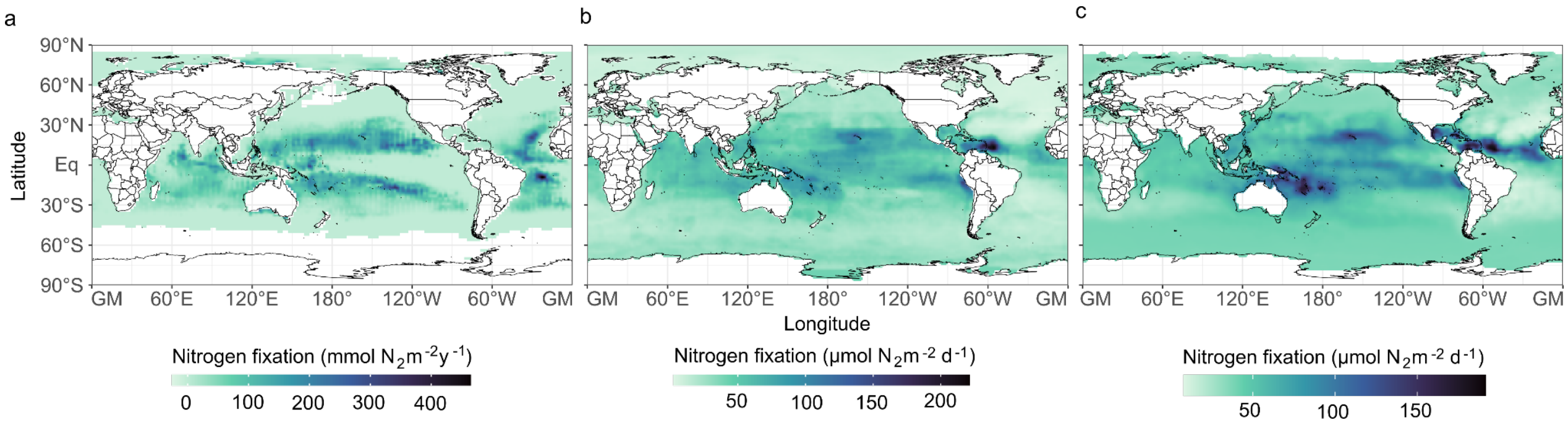

Supplementary Figure 8. Global distribution of climatological biological nitrogen fixation rates. Comparison of globally integrated nitrogen fixation estimates resampled to a consistent 1° grid for alignment with diazotroph richness model outputs. **a**, Wang et al.[2] (mmol N m−2 y−1), derived from an inverse biogeochemical and prognostic ocean model. **b**, Tang et al.[3] Random Forest (RF) based model and **c,** Tang et al.[3] Support Vector Regression (SVR) based model (μmol N m^{-2} d^{-1}). Daily rates were derived by integrating monthly climatologies.

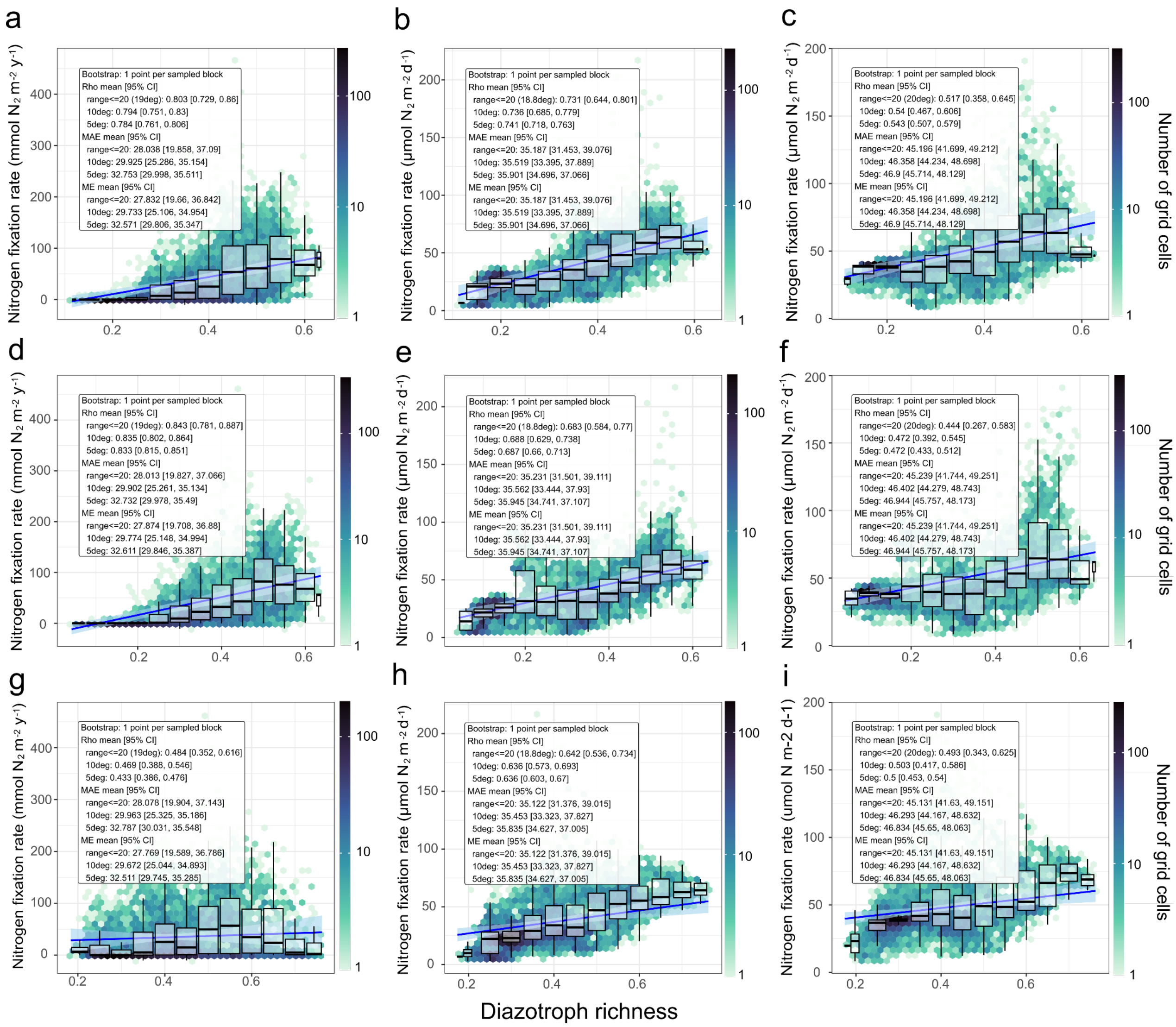

Supplementary Figure 9. Global relationships between predicted diazotroph richness and marine nitrogen fixation rates. The panels evaluate the biodiversity–ecosystem functioning relationship across three diazotroph richness groups and three independent nitrogen fixation products. **a–c**, Total annual normalized richness vs. **a** Wang et al.[2], **b** Tang et al.[3] Random Forest, and **c** Tang et al.[3] Support Vector Regression. **d–f**, Cyanobacterial richness vs. **d** Wang et al.[2], **e** Tang et al.[3] Random Forest, and **f** Tang et al.[3] Support Vector Regression. **g–i**, Non-cyanobacterial richness vs. **g** Wang et al.[2], **h** Tang et al.[3] Random Forest, and **i** Tang et al.[3] Support Vector Regression. Each panel displays the hexbin density of paired 1°×1° grid cell values, an ensemble linear fit mean (solid line), and +/- 1 standard deviation ribbon (shaded area) across richness realizations. Insets report one-point-per-

block bootstrap summaries for three spatial block scenarios: a model-specific variogram range capped at 20°, and fixed 10° and 5° blocks. Reported statistics include mean Spearman's ρ, Mean Absolute Error (MAE), and Mean Error (ME) with 95% confidence intervals derived from 1,000 bootstrap iterations.

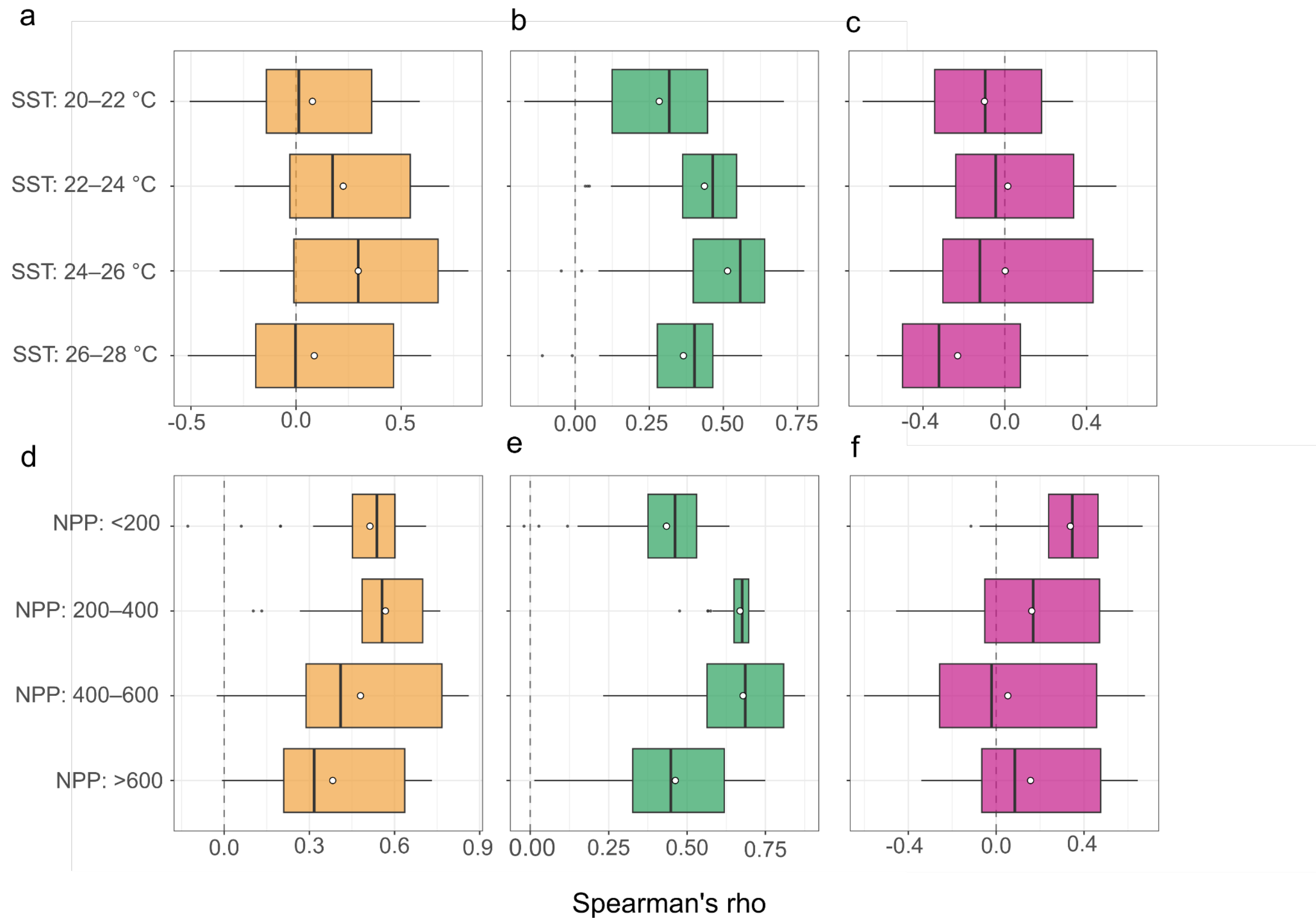

Supplementary Figure 10: Robustness of the diazotroph richness-nitrogen fixation relationship within temperature and productivity constrained strata. Boxplots show Spearman rank correlations between nitrogen fixation and richness for all richness realizations (n=90) after restricting analyses to sea surface temperature (SST) bins (panels **a-c**; 2°C increments: 20-22, 22-24, 24-26, and 26-28°C) for three richness datasets: **a**, total diazotroph richness; **b**, cyanobacterial diazotroph richness; and **c**, non-cyanobacterial diazotroph richness. This thermal window was selected as it encompasses the vast majority of global marine nitrogen fixation activity and the core niches of the most productive diazotrophic lineages, providing the most rigorous environment to test for non-thermal biological signals. Center lines indicate medians, boxes indicate interquartile ranges, whiskers indicate non-outlier ranges, and white points indicate means. Panels **d-f** show analyses across net primary production (NPP) bins (<200, 200-400, 400-600, and >600) again for **d** total, **e** cyanobacterial and **f** non-cyanobacterial richness. The vertical dashed

line at x=0 marks zero correlation. White points indicate the mean Spearman’s rho for each stratum.

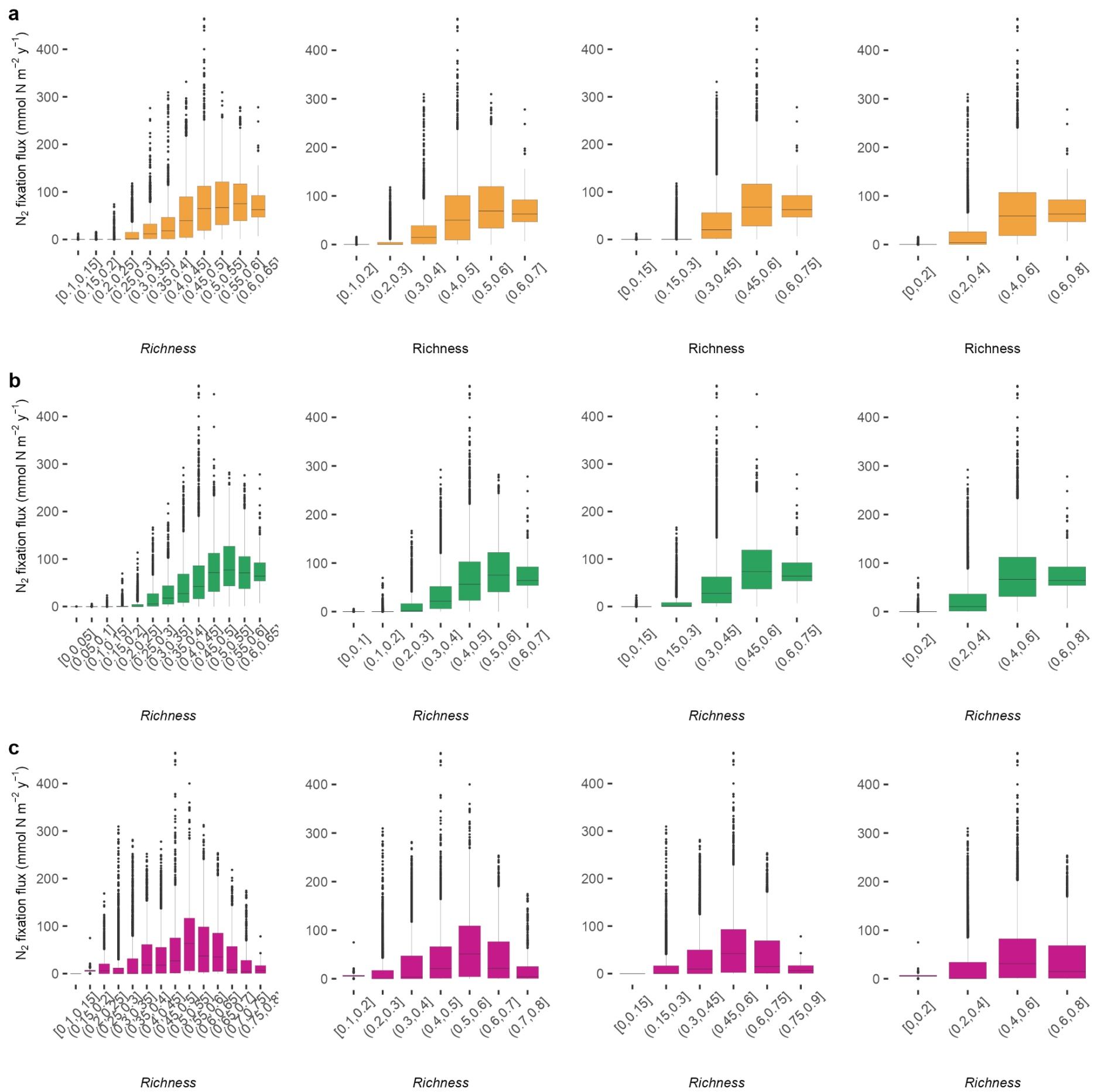

Supplementary Figure 11. Boxplots of diazotroph richness and nitrogen fixation rates across bin sizes. Boxplots showing increasing annual diazotroph richness bins on the x-axis and annual nitrogen fixation rates by Wang et al.[2] on the y-axis for **a** total, **b** cyanobacterial, and **c** non-cyanobacterial diazotroph communities.

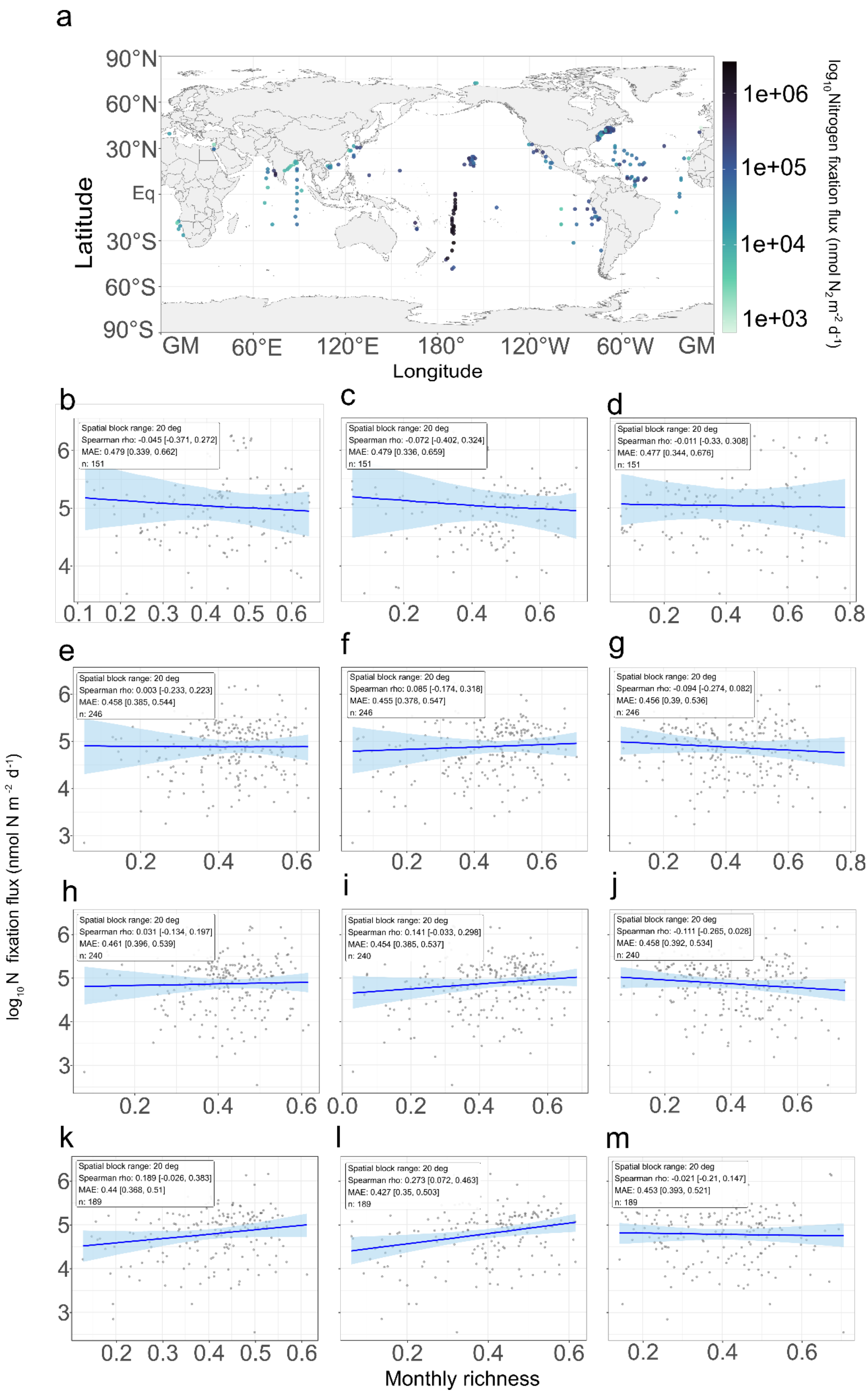

Supplementary Figure 12. Relationships between predicted diazotroph richness and

***in situ* marine nitrogen fixation rates. a** Global spatial distribution of 1° gridded samples containing *in situ* measured nitrogen fixation rates, compiled from Shao et al.[4], Raes et al.[5] and Chowdhury et al.[6]. Individual measurements were monthly averaged within each 1° x 1° grid cell to align with the spatial resolution of the richness models. Color gradients represent $\log_{10}$ transformed nitrogen fixation rates (nmol N m^{-2} d^{-1}). **b–m** Relationships between monthly *in situ* nitrogen fixation rates and modeled diazotroph richness across increasing spatial aggregation scales. The panels are organized by rows representing spatial resolutions, 1° (first row), 5° (second row), 10° (third row), and 20° (fourth row) and columns representing richness groups (Total, Cyanobacterial, and Non-cyanobacterial richness). For each grid cell, *in situ* measurements and modeled richness were monthly matched at the respective spatial resolution, with *in situ* values represented as the monthly mean. Points show individual grid-cell pairs. The solid blue line indicates the ensemble-mean linear fit, while the shaded ribbon shows the 95% spatial block bootstrap uncertainty band around the fitted relationship. Inset statistics report diagnostics from a variogram-informed spatial block-bootstrap (1,000 iterations; block range capped at 20°), including the Spearman correlation (ρ), mean absolute error (MAE), and the number of samples (n).

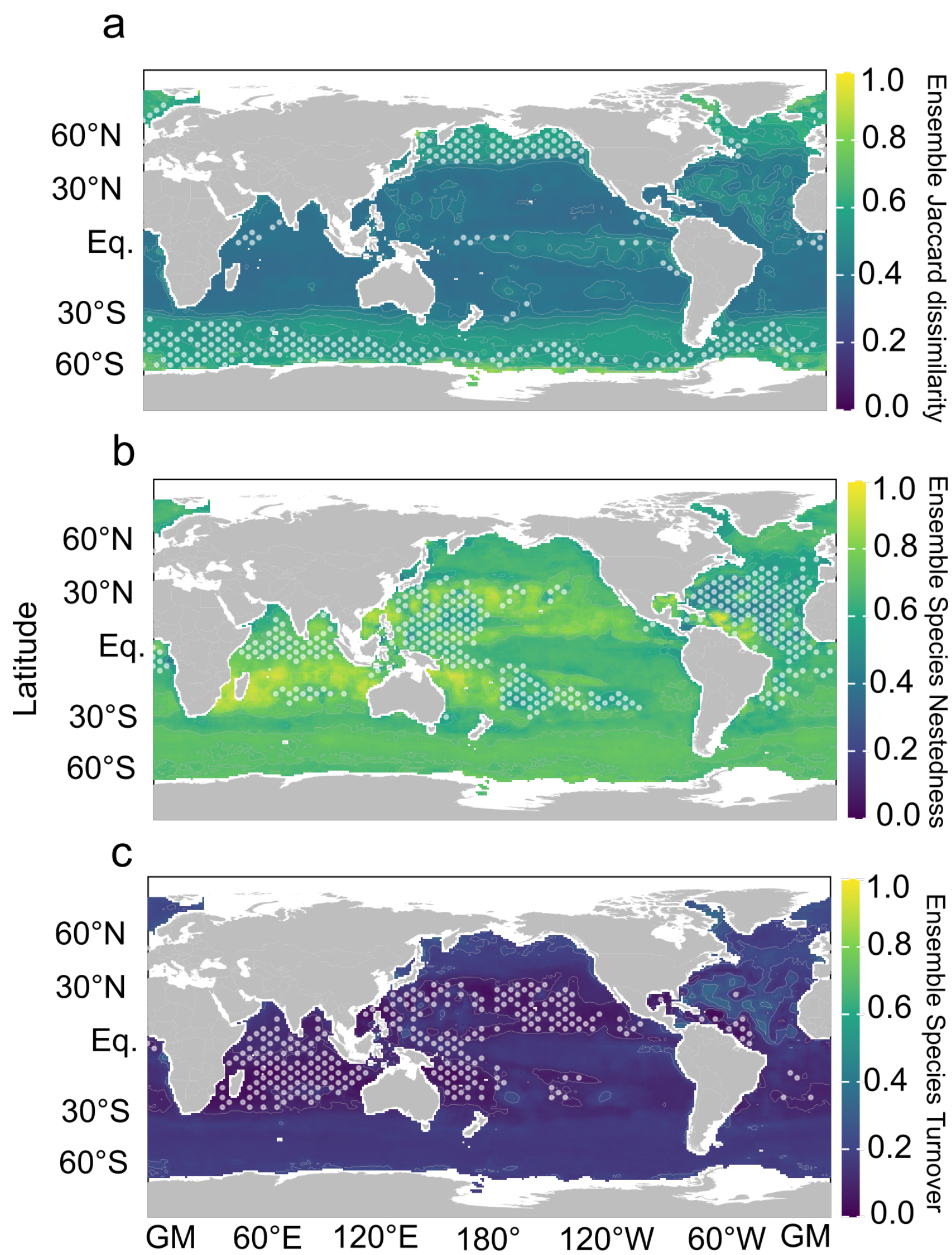

Supplementary Figure 13. Global patterns of beta diversity. Global maps of **a** annual Jaccard dissimilarity, **b** annual nestedness, and **c** annual species turnover. All three beta diversity indices were computed across 90 richness realizations, with each grid cell covering 1° longitude by 1° latitude for the total diazotroph community (n = 15). White stipples indicate areas where the coefficient of variation was above the 70th percentile, marking greater differences between richness realizations.

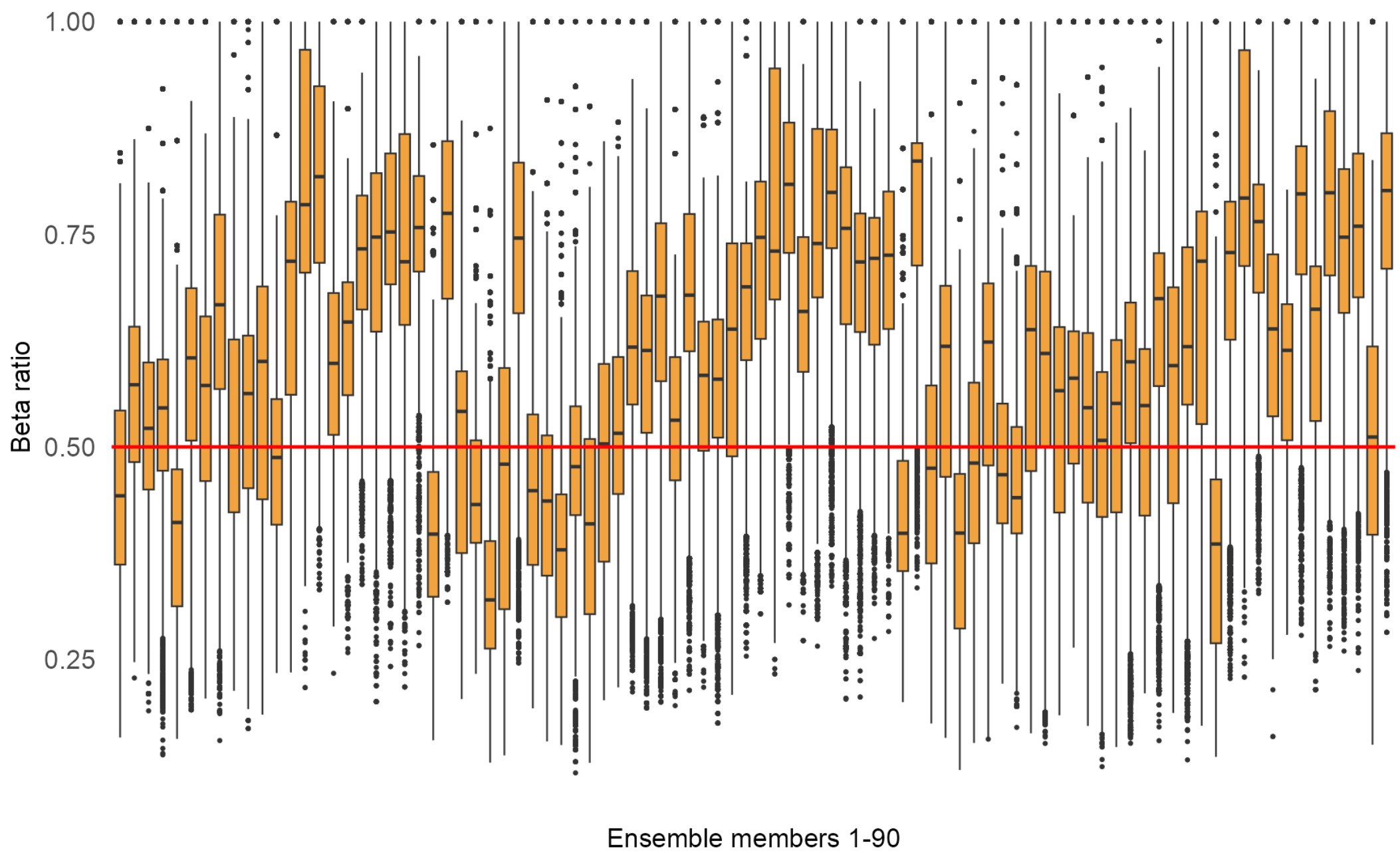

Supplementary Figure 14. Global annual beta ratios across ensemble members. Boxplots showing the range of beta ratio estimates (y-axis) across 90 ensemble members (x-axis) for a total of 15 modeled diazotrophs. The annual diazotroph beta ratio is computed as the ratio of nestedness over Jaccard dissimilarity with each grid cell covering 1° longitude by 1° latitude. The solid red line has been placed to visualize the threshold of either nestedness (> 0.5) or species turnover (< 0.5) being the dominant driver underlying the global richness pattern.

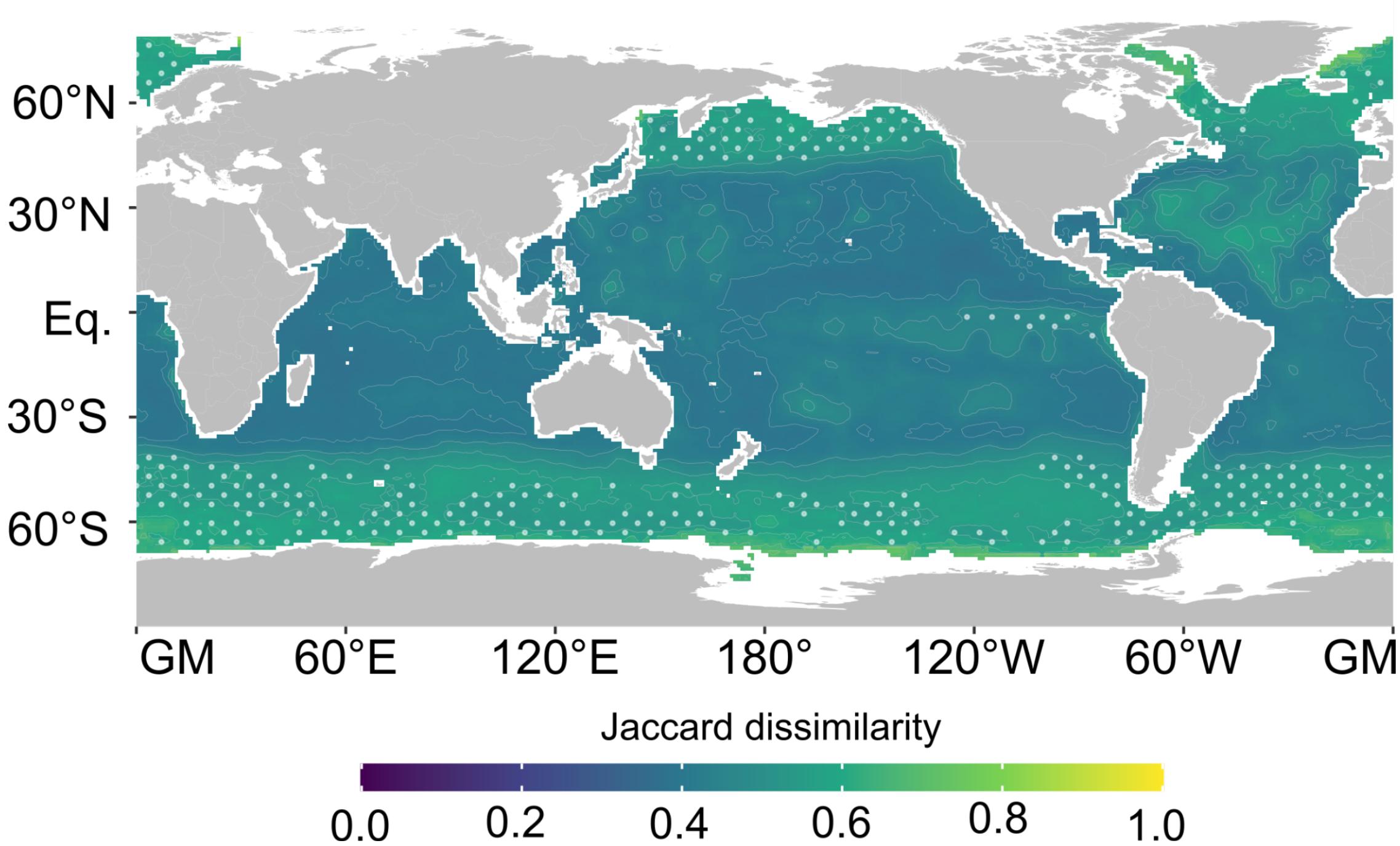

Supplementary Figure 15. Mean Jaccard dissimilarity per grid cell. Global maps of the annual Jaccard dissimilarity. The Jaccard dissimilarity for each grid cell is computed as the mean across all 12 monthly pairs. The Jaccard dissimilarity is computed as an ensemble across 90 ensemble members, with each grid cell covering 1° longitude by 1° latitude for the total diazotroph community (n = 15). We established the criterion for annual presence as the occurrence of at least one species within a grid cell for a minimum of one month over a twelve-month period. A value of zero indicates that the communities remain stable year-around, showing no change in community composition on a temporal (monthly) scale.

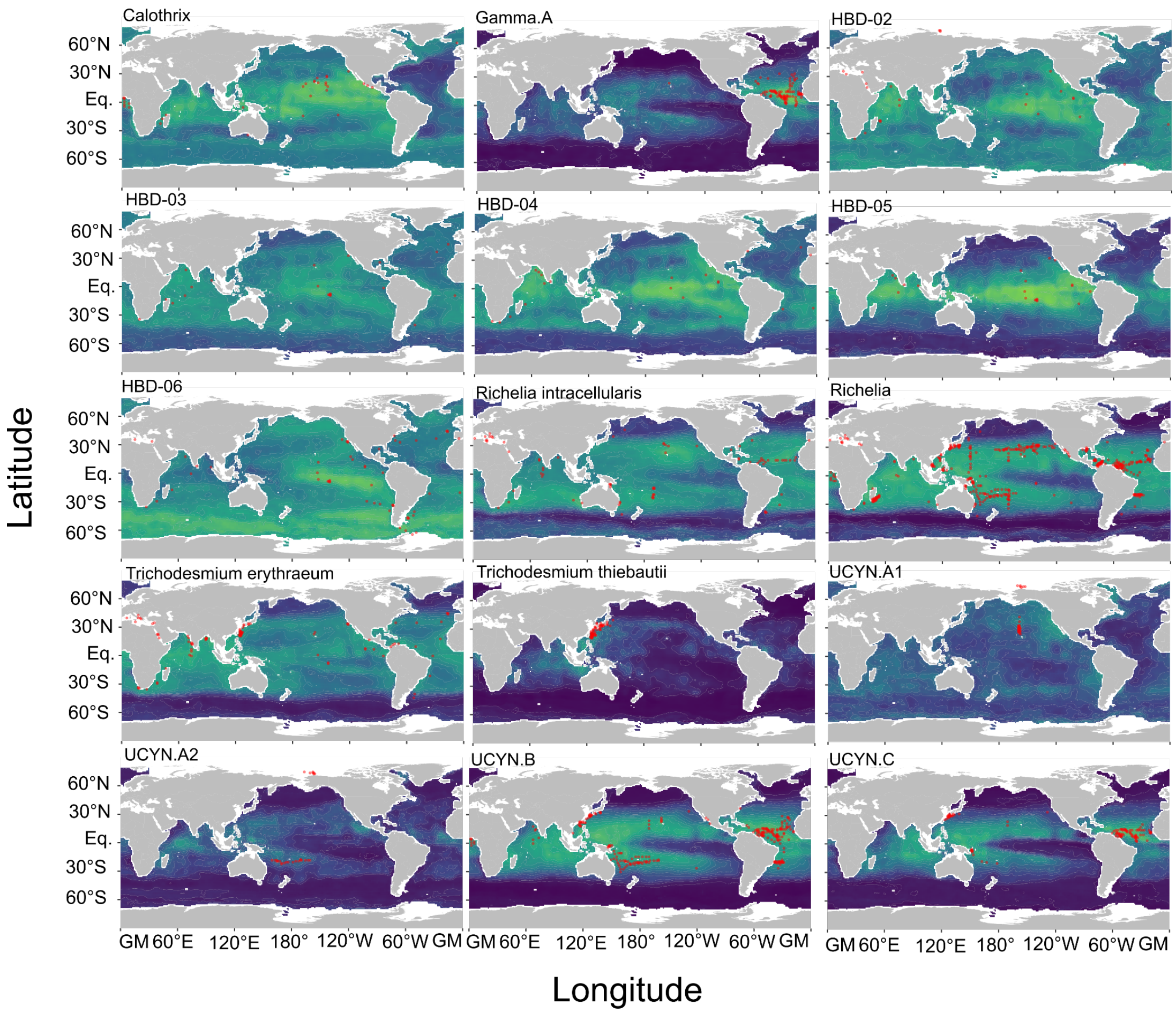

Supplementary Figure 16. Global diazotroph biogeographies. Individual spatial distributions of diazotrophs computed as the annual averaged habitat suitability per taxon per area across 12 months. Each taxon was modeled separately for each month on a 1° longitude by 1° latitude spatial resolution. Red points indicate the gridded presences that were available for the modeling pipeline.

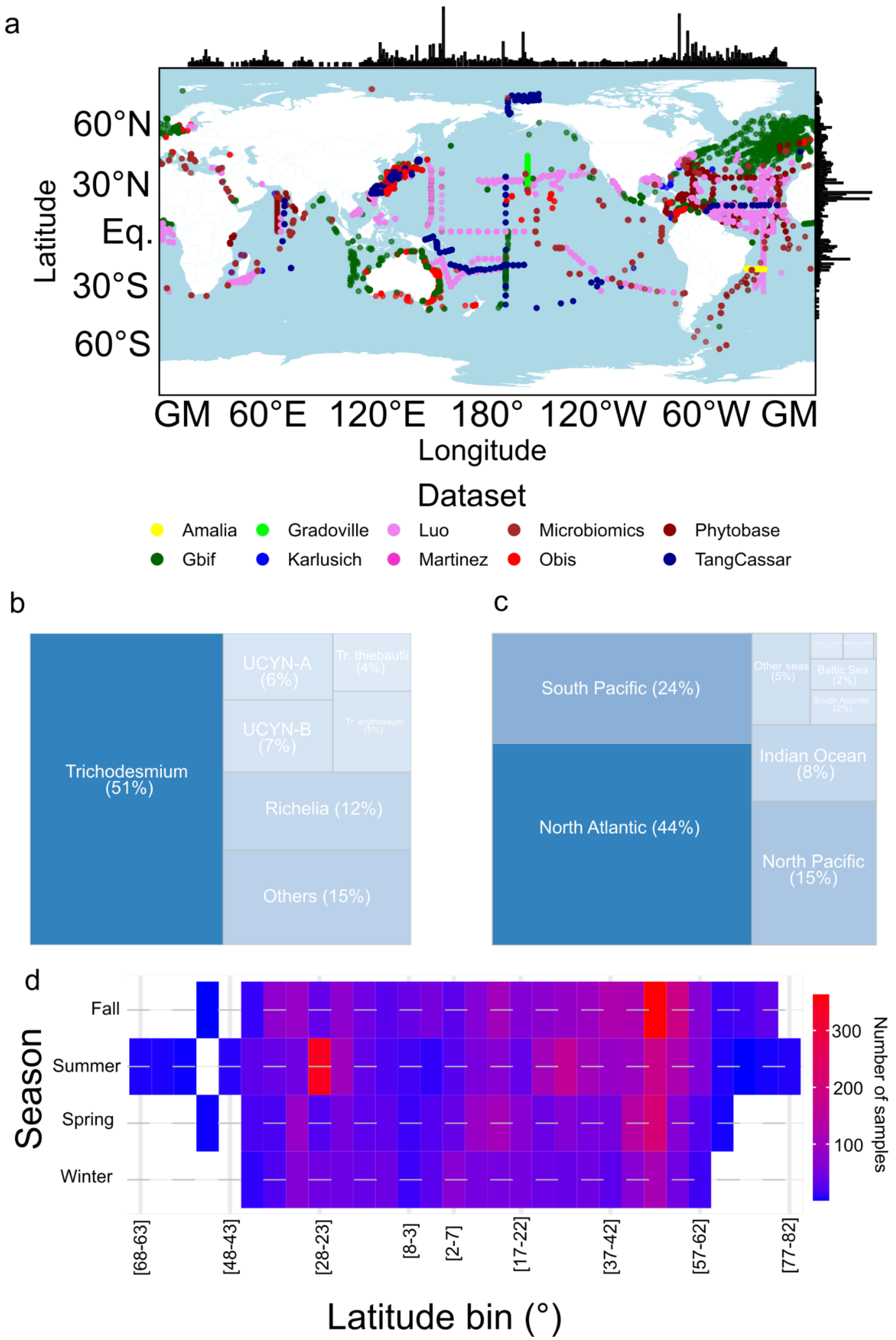

Supplementary Figure 17. Global sampling effort. a Global map of diazotroph presence records (n > 22,000) with longitudinal and latitudinal marginal histograms colored by sources (yellow: Detoni et al.[7], green: Gradoville et al.[8], pink: Luo et al.[9], brown: Ocean

Microbiomics Database[1], dark red: Phytobase[10], dark green: GBIF, blue: Karlusich et al.[11], dark pink: Martínez-Pérez et al.[12], red: OBIS, dark blue: Tang and Cassar[13]. **b** A treemap showing the percentage fraction of individual diazotroph taxa. Percentages decrease along light to dark blue color gradients. **c** A treemap showing the fraction of total observations/sampling effort in percentage for each ocean basin. Percentages decrease along light-to-dark blue color gradients. The Southern Ocean is not shown, as the fractions of observational records falling into this region is below 1%. **d** Seasonal sampling coverage visualized by sampling events, defined via latitudinal bins (binning size 5°) and seasons.

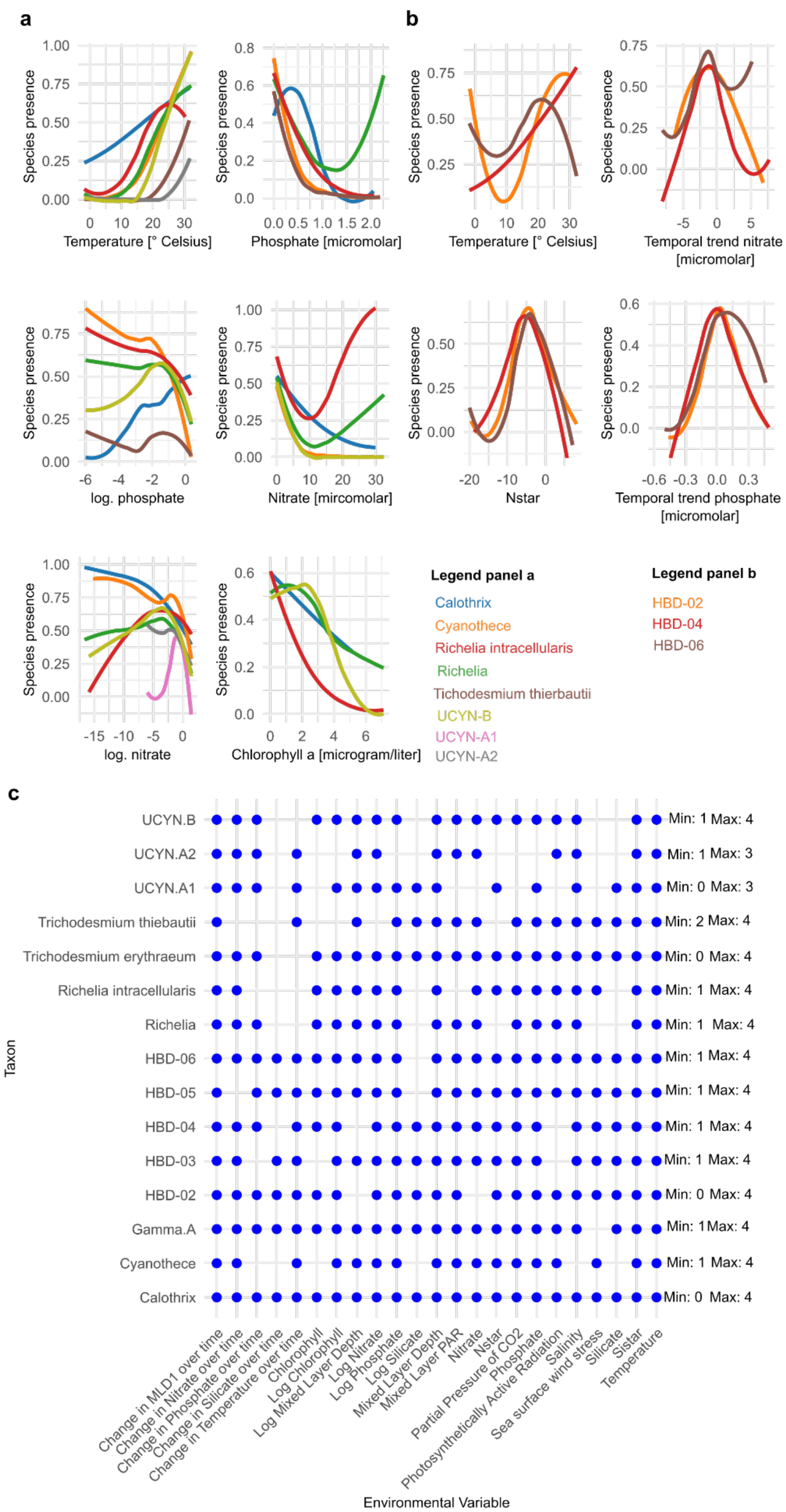

Supplementary Figure 18. Environmental response curves and predictor ensembles. Selected response curves fitted for **a** cyanobacterial and **b** non-cyanobacterial diazotrophs across top-ranked environmental predictors based on the General Additive Model. Response curves have been averaged across all background selection strategies. **c** Overview of variables included in the final ensemble models for each diazotroph. The x-axis represents

the predictor variables and the y-axis lists the diazotrophs modeled. On the right, annotations indicate the minimum and maximum number of predictors used across all ensemble members for each diazotroph. A zero means the maximum has been used throughout all ensemble members.

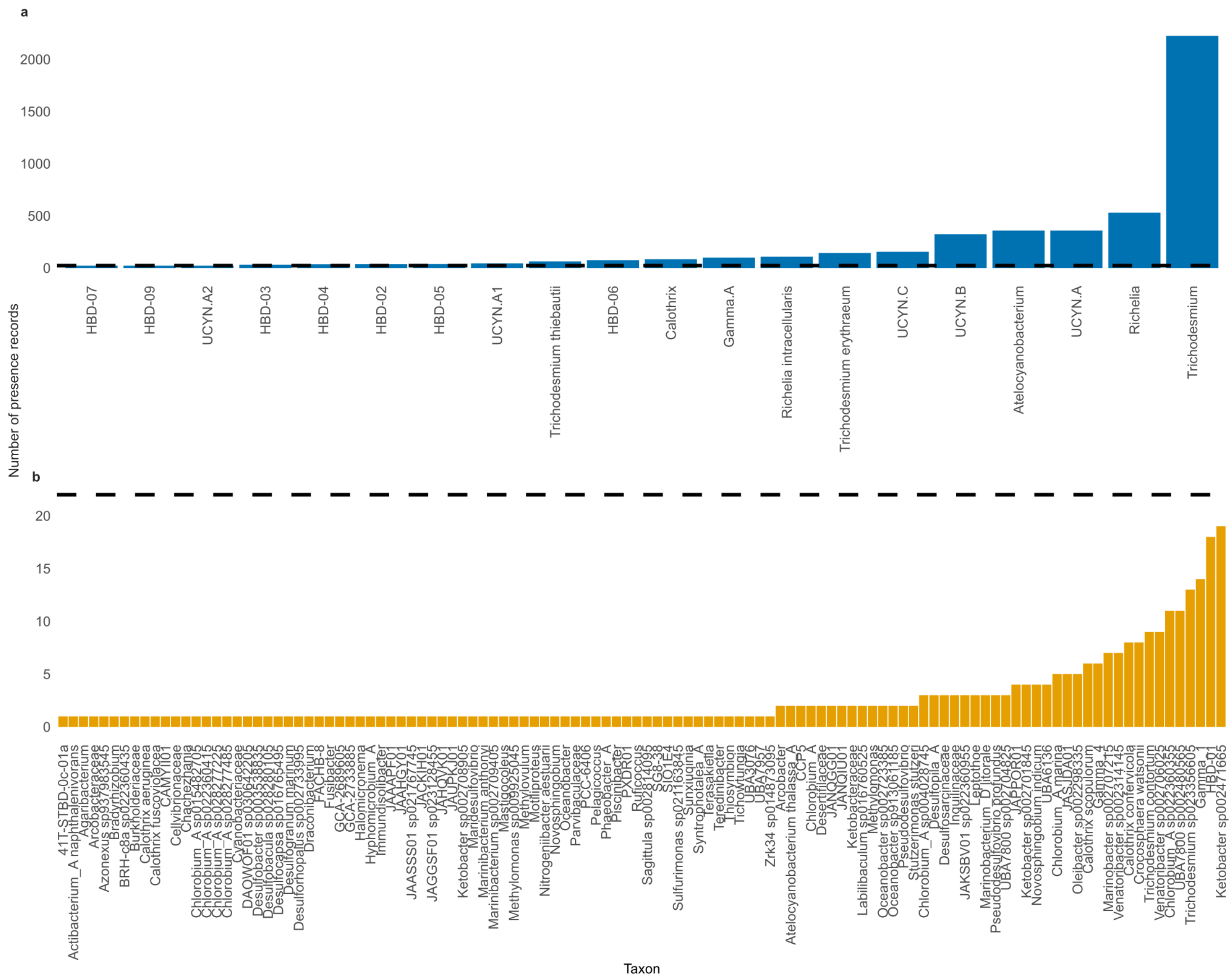

Supplementary Figure 19. Number of presence records by taxa. a Barplots showing the number of presence records for each taxon with a black dashed line indicating the lower threshold of 22 observations, which were used for modeling. **b** shows the remaining diazotrophs in our data collection displaying less than 22 observations, with the majority showing less than 5 observations across all samples.

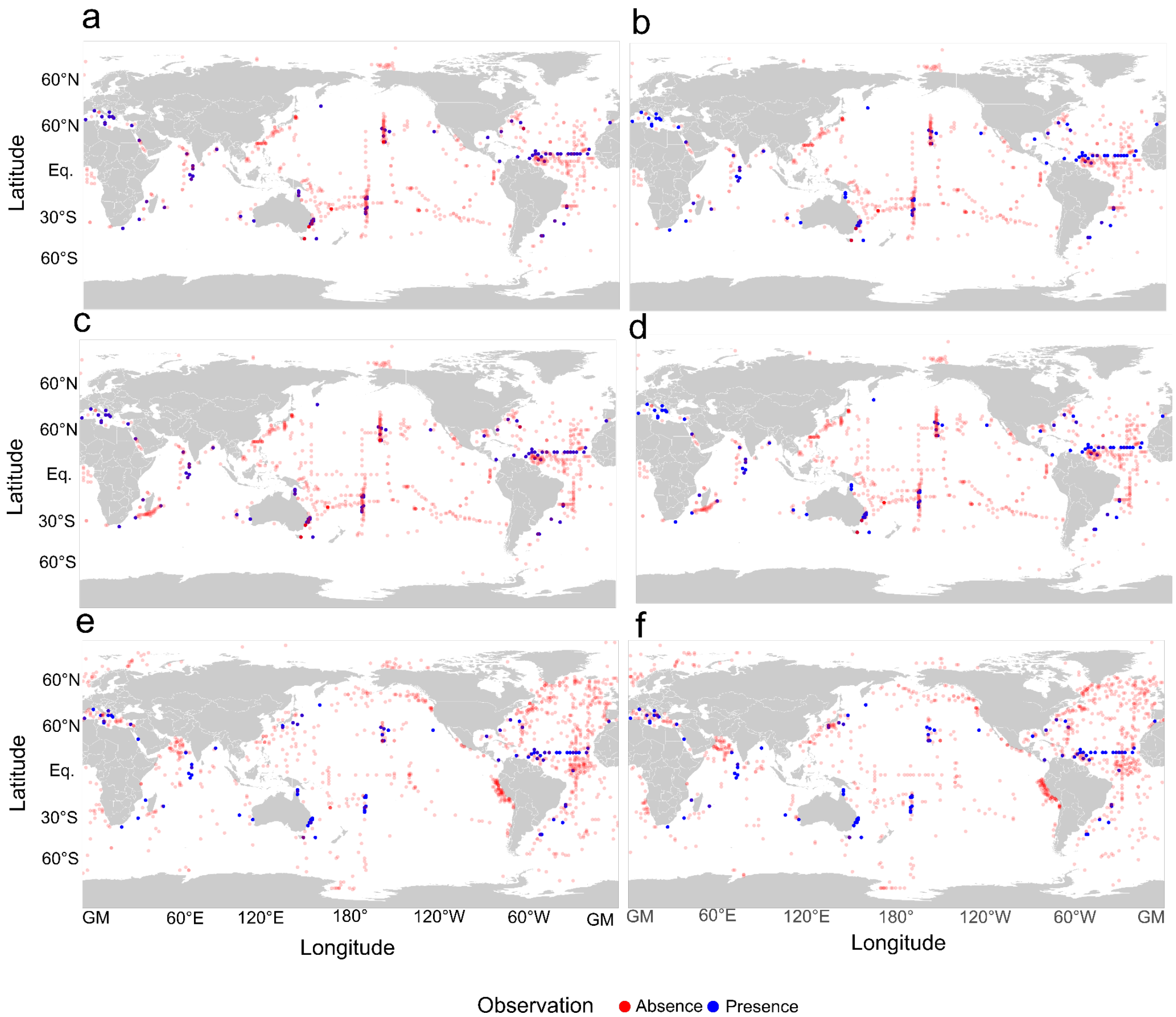

Supplementary Figure 20. Global map visualizing background data selection via six strategies for one exemplary taxon. Global distributions of presences (blue circles) and pseudo-absences (red circles) for *Richelia intracellularis* across varying background selection strategies. **a-b** cruise-specific, **c-d** group-specific, and **e-f** total background selection. Each background selection can either be overlapping (left column panels) or non-overlapping (right column panels), with the former allowing sampling of pseudo-absences at locations where presences have been detected.

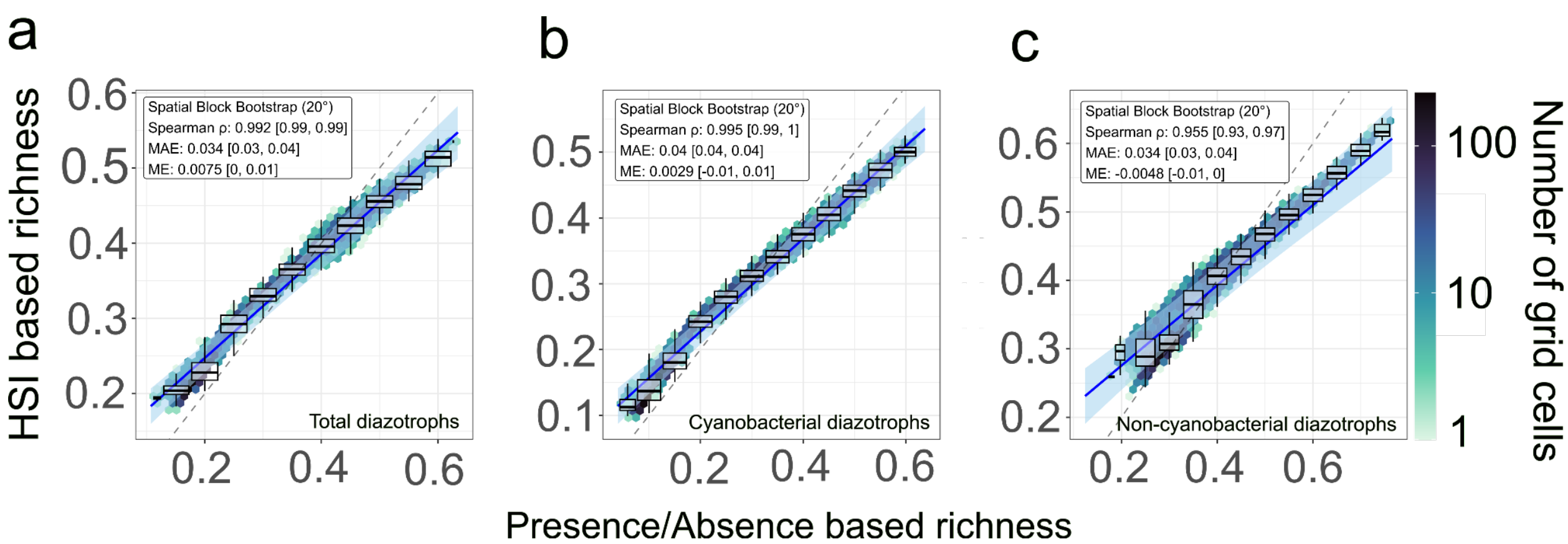

Supplementary Figure 21. Spatial block bootstrap analysis comparing presence/absence-based and Habitat Suitability Index based diazotroph richness. Panels show total **a**, cyanobacterial **b**, and non-cyanobacterial **c** richness groups using paired, normalized annual richness values. These values were extracted at corresponding coordinates and computed either from monthly averaged and normalized richness estimates based on the Habitat Suitability Index (y-axis) or presence-absence-based richness (x-axis). Hexagons represent the density of paired observations (number of grid cells). Overlaid boxplots summarize the distribution of presence-absence-based richness values within fixed richness bins (width = 0.05). The blue line and shaded ribbon indicate the ensemble mean linear fit and ± 1 standard deviation across richness realizations (n = 90). Insets report spatial block-bootstrap diagnostics based on variogram-informed block sizes: Spearman's rank correlation (ρ), mean absolute error (MAE), and mean error (ME), each shown as the median with 95% confidence intervals from 1,000 bootstrap iterations.

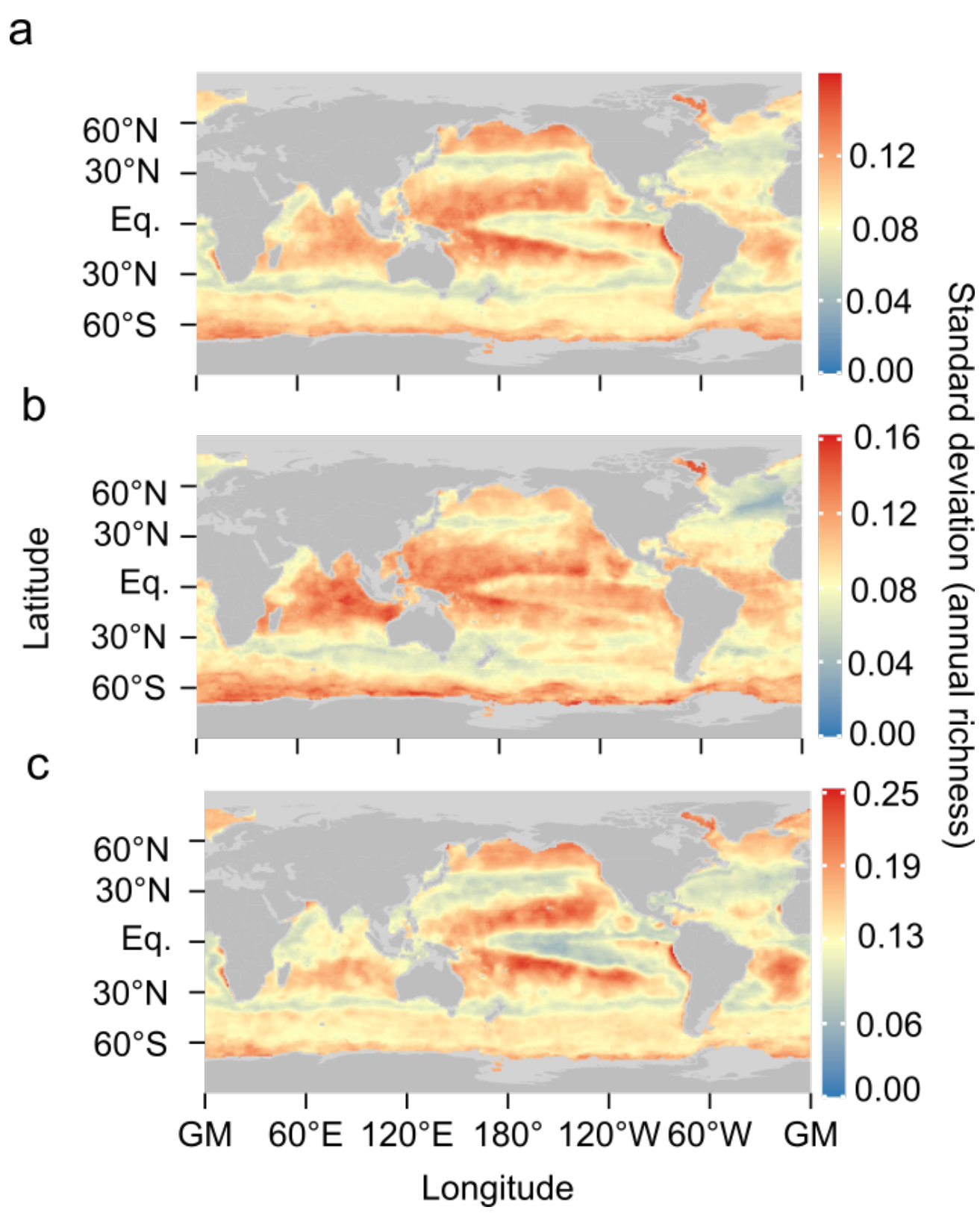

Supplementary Figure 22. Model uncertainty for derived diazotroph richness estimates. Standard deviation of normalized annual richness predictions is shown for **a** total, **b** cyanobacterial, and **c** non-cyanobacterial richness. Maps were generated using ensemble predictions from presence-absence models, and spatial variability was computed as the grid cell-wise standard deviation across richness realizations.

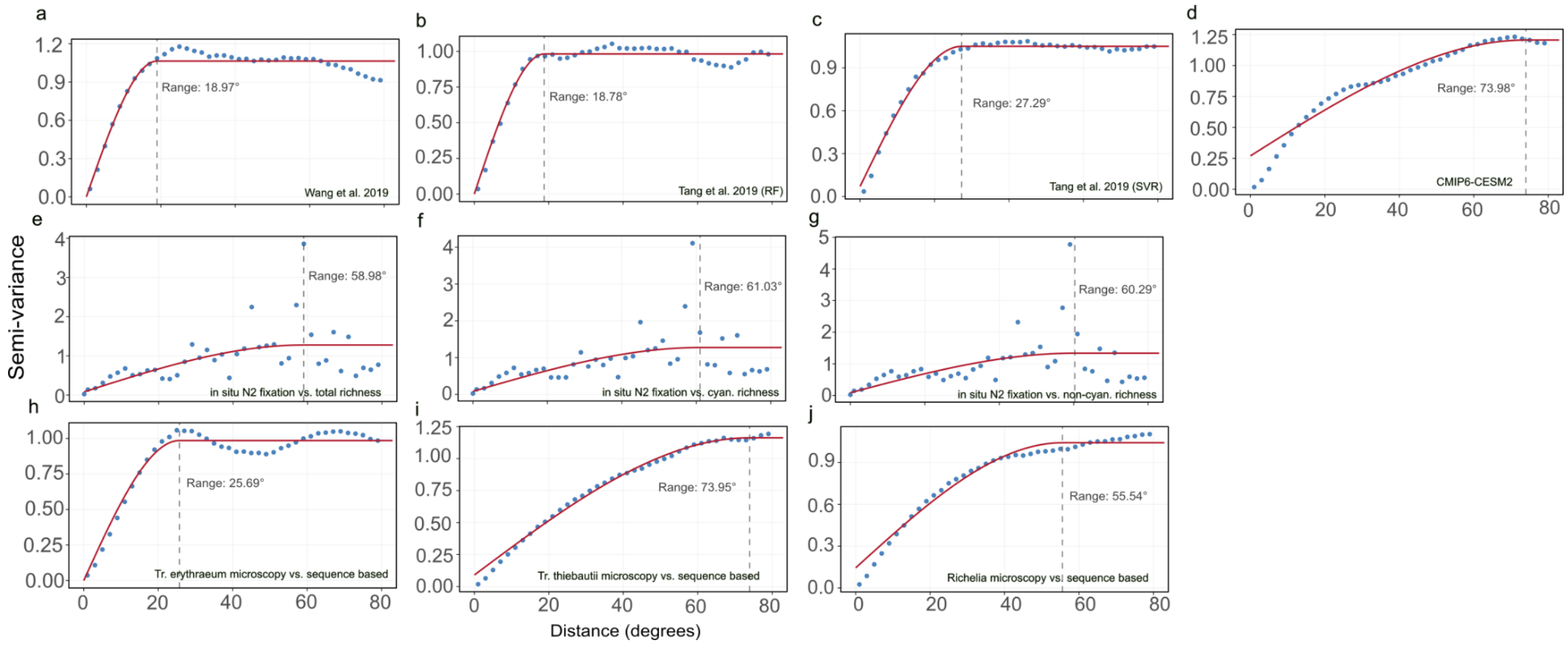

Supplementary Figure 23. Residual semi-variograms for all correlation pairs. The figure combines the residual based biodiversity-ecosystem function global nitrogen fixation relationships **a-d**, the residual-based biodiversity ecosystem function *in situ* nitrogen fixation relationships **e-g**, and the microscopy vs. sequence-based habitat suitability relationships **h-j**. Blue points represent empirical semivariance of the residuals, red lines represent fitted spherical variogram models, and dashed vertical lines indicate the fitted effective range in degrees.

Supplementary Table S1.

Supplementary table showing mean ± SD explanatory skill used for the environmental predictor ranking for GLM (D2), GAM (adjusted R²), and RF (Out-Of-Bag Error) across all six background selection strategies. Environmental variables: T (sea surface temperature, °C), Sal (sea surface salinity), N (nitrate, µM), P (phosphate, µM), Si (silicic acid, µM), MLD (mixed layer depth, meters), PAR (photosynthetically active radiation, µmol $m^{-2}s^{-1}$), Chl (chlorophyll, µg l^{-1}), Wind.CCMP (sea surface wind stress, m s^{-1}), pCO_2 (carbon dioxide partial pressure in the surface sea, µatm), MLPAR (photosynthetically available radiation over the mixed layer depth, µmol m^{-2} s^{-1}), Nstar (excess concentration of nitrate in relation to the çedfield ratio, µM), Sistar (the ratio of nitrate to silicic acid, µM), dT_dt (temporal trends of sea surface temperature, °C), dN_dt (temporal trends of nitrate, µM), dP_dt (temporal trends of phosphate, µM), dSi_dt (temporal trends of silicate, µM), dMLD_dt (temporal trends of mixed layer depth, meters).

Taxon	Statistic	T	Sal	N	P	Si	MLD	PAR	Chl	Wind CCMP	pCO2	MLPAR	Nstar	Sistar	dT_dt	dN_dt	dP_dt	dSi_dt	dMLD_dt
Calothrix	1-OOB.error	0.69	0.69	0.65	0.65	0.64	0.63	0.66	0.66	0.65	0.62	0.64	0.68	0.64	0.61	0.66	0.61	0.61	0.67
Calothrix	adj.Dsq	0.07	0.23	0.03	0.08	0.01	0.03	0.04	0.01	0.04	0.03	0.06	0.12	0.02	0.02	0.01	0.02	0.02	0.03
Calothrix	adj.Rsq	0.12	0.23	0.03	0.08	0.01	0.02	0.04	0.04	0.06	0.03	0.08	0.14	0.02	0.03	0.01	0.02	0.01	0.07
Cyanothece	1-OOB.error	0.7	0.61	0.62	0.65	0.6	0.66	0.7	0.66	0.68	0.68	0.65	0.62	0.64	0.65	0.65	0.63	0.61	0.64
Cyanothece	adj.Dsq	0.25	0.03	0.19	0.17	0.03	0.15	0.07	0.02	0.04	0.17	0.16	0.07	0.15	0.05	0.14	0.04	0.01	0.06
Cyanothece	adj.Rsq	0.23	0.03	0.16	0.15	0.03	0.14	0.06	0.03	0.16	0.19	0.17	0.08	0.14	0.07	0.15	0.05	0.03	0.08
Gamma.A	1-OOB.error	0.61	0.64	0.58	0.6	0.58	0.61	0.65	0.6	0.57	0.6	0.59	0.58	0.61	0.59	0.56	0.57	0.55	0.61
Gamma.A	adj.Dsq	0.14	0.19	0.12	0.22	0.08	0.05	0.15	0.05	0.03	0.12	0.22	0.16	0.08	0.15	0.08	0.1	0.06	0.02
Gamma.A	adj.Rsq	0.13	0.19	0.1	0.22	0.08	0.05	0.17	0.05	0.05	0.12	0.24	0.15	0.11	0.15	0.08	0.1	0.1	0.02
HBD-02	1-OOB.error	0.62	0.64	0.58	0.6	0.61	0.61	0.59	0.57	0.57	0.62	0.58	0.59	0.65	0.6	0.62	0.6	0.63	0.61
HBD-02	adj.Dsq	0.03	0.03	0.01	0.03	0.01	0.02	0.01	0.02	0.03	0.03	0.03	0.08	0.01	0.04	0.03	0.04	0.01	0.01
HBD-02	adj.Rsq	0.06	0.04	0	0.05	0.02	0.02	0.02	0.02	0.03	0.03	0.04	0.08	0.01	0.03	0.03	0.06	0.02	0
HBD-03	1-OOB.error	0.62	0.62	0.59	0.59	0.65	0.62	0.57	0.57	0.57	0.59	0.63	0.63	0.7	0.62	0.6	0.57	0.63	0.59
HBD-03	adj.Dsq	0.06	0.04	0.04	0.07	0.06	0.05	0.02	0.03	0.01	0.04	0.02	0.06	0.02	0.03	0.1	0.01	0	0.02
HBD-03	adj.Rsq	0.05	0.06	0.03	0.06	0.04	0.05	0.01	0.02	0.08	0.04	0.01	0.06	0.01	0.02	0.09	0.02	0.05	0.01
HBD-04	1-OOB.error	0.63	0.63	0.64	0.59	0.62	0.58	0.6	0.58	0.63	0.6	0.61	0.66	0.63	0.61	0.64	0.6	0.61	0.57
HBD-04	adj.Dsq	0.05	0.02	0.05	0.08	0.04	0.01	0.01	0.03	0.04	0.05	0.01	0.07	0.03	0.05	0.06	0.02	0	0.02
HBD-04	adj.Rsq	0.05	0.01	0.04	0.08	0.04	0.01	0	0.02	0.04	0.05	0.03	0.07	0.03	0.05	0.06	0.02	0	0.01
HBD-05	1-OOB.error	0.71	0.64	0.65	0.6	0.59	0.62	0.62	0.56	0.64	0.66	0.64	0.68	0.67	0.63	0.61	0.62	0.62	0.65
HBD-05	adj.Dsq	0.17	0.06	0.05	0.09	0.03	0.01	0.07	0.07	0.07	0.2	0.06	0.15	0.02	0.06	0.02	0.01	0	0.05
HBD-05	adj.Rsq	0.16	0.06	0.05	0.09	0.02	0.01	0.05	0.06	0.08	0.2	0.12	0.15	0.02	0.06	0.02	0.01	0	0.04
HBD-06	1-OOB.error	0.61	0.6	0.6	0.59	0.54	0.6	0.56	0.55	0.56	0.55	0.52	0.59	0.6	0.62	0.64	0.57	0.55	0.59
HBD-06	adj.Dsq	0.02	0	0.03	0.03	0.01	0.03	0.01	0.03	0.03	0.01	0.02	0.03	0.02	0.01	0.02	0.02	0	0.01
HBD-06	adj.Rsq	0.04	0.01	0.04	0.03	0.01	0.03	0.01	0.03	0.03	0.01	0.02	0.04	0.03	0.02	0.06	0.02	0.01	0.01
Richelia intrac	1-OOB.error	0.66	0.68	0.61	0.62	0.61	0.64	0.67	0.62	0.62	0.63	0.61	0.6	0.62	0.64	0.64	0.61	0.63	0.62
Richelia intrac	adj.Dsq	0.09	0.03	0.08	0.06	0.02	0.04	0	0.03	0.02	0.01	0.01	0.03	0.07	0.01	0.05	0.02	0	0.05
Richelia intrac	adj.Rsq	0.12	0.02	0.08	0.07	0.02	0.04	0.05	0.04	0.06	0.02	0.01	0.03	0.07	0.03	0.05	0.04	0.01	0.05
Richelia	1-OOB.error	0.62	0.58	0.59	0.56	0.55	0.62	0.58	0.57	0.55	0.6	0.55	0.54	0.58	0.53	0.58	0.55	0.54	0.61
Richelia	adj.Dsq	0.18	0	0.13	0.08	0.01	0.05	0.06	0.04	0.03	0.07	0.06	0.02	0.11	0	0.08	0.03	0	0.07
Richelia	adj.Rsq	0.19	0.04	0.13	0.08	0.01	0.05	0.08	0.08	0.04	0.08	0.07	0.02	0.12	0.02	0.09	0.04	0.01	0.08
Trichodesmiur	1-OOB.error	0.66	0.66	0.65	0.65	0.64	0.68	0.65	0.65	0.65	0.65	0.64	0.65	0.67	0.65	0.66	0.65	0.63	0.68
Trichodesmiur	adj.Dsq	0.11	0.01	0.07	0.04	0.02	0.03	0.01	0.03	0.04	0.04	0.01	0.04	0.09	0.01	0.07	0.02	0.01	0.05
Trichodesmiur	adj.Rsq	0.12	0.03	0.06	0.04	0.02	0.03	0.05	0.03	0.04	0.04	0.02	0.03	0.08	0.01	0.07	0.04	0.01	0.07
Trichodesmiur	1-OOB.error	0.78	0.85	0.76	0.77	0.77	0.8	0.77	0.71	0.8	0.78	0.82	0.75	0.84	0.79	0.74	0.73	0.71	0.79
Trichodesmiur	adj.Dsq	0.34	0.46	0.26	0.27	0.16	0.37	0.24	0.1	0.38	0.24	0.45	0.17	0.28	0.12	0.2	0.19	0.02	0.21
Trichodesmiur	adj.Rsq	0.31	0.55	0.25	0.28	0.39	0.4	0.34	0.1	0.41	0.28	0.49	0.22	0.53	0.13	0.22	0.23	0.06	0.22
UCYN.B	1-OOB.error	0.61	0.56	0.56	0.53	0.51	0.6	0.54	0.54	0.52	0.56	0.53	0.53	0.55	0.54	0.56	0.51	0.51	0.57
UCYN.B	adj.Dsq	0.25	0.02	0.12	0.1	0.02	0.06	0.06	0.04	0.01	0.11	0.07	0.04	0.07	0.03	0.09	0.05	0	0.05
UCYN.B	adj.Rsq	0.24	0.04	0.11	0.1	0.01	0.08	0.07	0.07	0.02	0.13	0.08	0.05	0.08	0.04	0.09	0.05	0	0.05

Supplementary Table S2.

Environmental parameters that have been selected to reflect oceanic conditions that shape species' distributions via effects on physiology, growth, or species competition. Variables are available at a monthly (n = 12) climatological and globally gridded resolution (1° latitude × 1° longitude).

Candidate predictor	Variable nickname	Unit	Source
Sea Surface Temperature	SST	degree Celsius	World Ocean Atlas
Salinity	S	Practical salinity units	World Ocean Atlas
Nitrate	NO3-	µM	World Ocean Atlas
Phosphate	PO43-	µM	World Ocean Atlas
Silicic acid	Si(OH)4	µM	World Ocean Atlas
Mixed layer depth	MLD	meters	de Boyer Montégut 2004
Photosynthetically active radiation	PAR	µmol m-2 s-1	Seas-Viewing Wide Field-of-view Sensor
Chlorophyll	Chl	µg liter-1	Seas-Viewing Wide Field-of-view Sensor
Sea surface wind stress	SSW	m s-1	Corss-Calibrated Multi-Platform
Carbon dioxide partial pressure	pCO2	µatm	Landschützer, Gruber Bakker 2015
Photosynthetically active radiation over the mixed layer depth	MLPAR	µmol m-2 s-1	Brun et al., 2015
Excess concentration of Nitrate to the Redfield Ration	Nstar	µM	[Nitrate] - 16*[Phsophate]
Ratio of silicic acid to nitrate	SiStar	µM	[Silicate]/[Nitrate]
Temporal trends of sea surface temperature	dT/dt	degree Celsius month-1	Self computed
Temporal trends of nitrate	dNO3-/dt	µM month-1	Self computed
Temporal trends of phosphate	dNPO43-/dt	µM month-1	Self computed
Temporal trends of mixed layer depth	dMLD/dt	µM month-1	Self computed
Logarithmic nitrate concentration to the base of ten	logNO3-		Self computed
Logarithmic phosphate concentration to the base of ten	logPO43-		Self computed
Logarithmic silicate concentration to the base of ten	logSi(OH)4		Self computed

References

1. Paoli, L. *et al.* Biosynthetic potential of the global ocean microbiome. *Nature* **607**, 111–118 (2022).
2. Wang, W.-L., Moore, J. K., Martiny, A. C. & Primeau, F. W. Convergent estimates of marine nitrogen fixation. *Nature* **566**, 205–211 (2019).
3. Tang, W., Li, Z. & Cassar, N. Machine Learning Estimates of Global Marine Nitrogen Fixation. *J. Geophys. Res. Biogeosciences* **124**, 717–730 (2019).
4. Shao, Z. *et al.* Global oceanic diazotroph database version 2 and elevated estimate of global oceanic N_2 fixation. *Earth Syst. Sci. Data* **15**, 3673–3709 (2023).
5. Raes, E. J. *et al.* N2 Fixation and New Insights Into Nitrification From the Ice-Edge to the Equator in the South Pacific Ocean. *Front. Mar. Sci.* **7**, 389 (2020).
6. Chowdhury, S. *et al.* Diazotrophy in the Indian Ocean: Current understanding and future perspectives. *Limnol. Oceanogr. Lett.* **8**, 707–722 (2023).
7. Detoni, A. M. S., Subramaniam, A., Haley, S. T., Dyhrman, S. T. & Calil, P. H. R. Cyanobacterial Diazotroph Distributions in the Western South Atlantic. *Front. Mar. Sci.* **9**, 856643 (2022).
8. Gradoville, M. R. *et al.* Latitudinal constraints on the abundance and activity of the cyanobacterium UCYN-A and other marine diazotrophs in the North Pacific. *Limnol. Oceanogr.* **65**, 1858–1875 (2020).
9. Luo, Y.-W. *et al.* Database of diazotrophs in global ocean: abundance, biomass and nitrogen fixation rates. *Earth Syst. Sci. Data* **4**, 47–73 (2012).
10. Righetti, D., Vogt, M., Zimmermann, N. E., Guiry, M. D. & Gruber, N. PhytoBase: A global synthesis of open-ocean phytoplankton occurrences. *Earth Syst. Sci. Data* **12**, 907–933 (2020).
11. Pierella Karlusich, J. J. *et al.* Global distribution patterns of marine nitrogen-fixers by imaging and molecular methods. *Nat. Commun.* **12**, 4160 (2021).
12. Martínez-Pérez, C. *et al.* The small unicellular diazotrophic symbiont, UCYN-A, is a key player in the marine nitrogen cycle. *Nat. Microbiol.* **1**, 16163 (2016).
13. Tang, W. & Cassar, N. Data-Driven Modeling of the Distribution of Diazotrophs in the Global Ocean. *Geophys. Res. Lett.* **46**, 12258–12269 (2019).
